# Supplementary material for: An FGFR1-SPRY2 Signaling Axis Limits Basal Cell Proliferation in the Steady-State Airway Epithelium
Source: Dev Cell. 2016 Apr 4;37(1):85–97. doi: 10.1016/j.devcel.2016.03.001 (PMC4825408; doi:10.1016/j.devcel.2016.03.001)
Supplement: Document S1. Supplemental Experimental Procedures and Figures S1–S6 [file mmc1.pdf]

Developmental Cell, Volume *37*

Supplemental Information

An FGFR1-SPRY2 Signaling Axis Limits Basal Cell Proliferation in the Steady-State Airway Epithelium

Gayan I. Balasooriya, Jo-Anne Johnson, M. Albert Basson, and Emma L. Rawlins

Supplemental Information Inventory

Figure S1, related to Figure 1. *Fgfr1* and *Spry2* are expressed in tracheal basal cells.

Figure S2, related to Figures 1 and 2. *Fgfr1* heterozygous basal cells display a similar phenotype to conditional knock-out basal cells.

Figure S3, related to Figures 3 and 6. Recombination of $Fgfr1^{fx}$ and $Spry2^{fx}$ in vitro.

Figure S4, related to Figure 3. *Fgfr1* cKO basal cells are more resistant to EGFR signalling inhibition.

Figure S5, related to Figure 3. FGF2 stimulation of wild-type basal cells results in a small drop in pERK1/2 levels.

Figure S6, related to Figure 5. Ciliated cell differentiation and epithelial organisation are normal in the *Spry2* conditional knock-outs.

Supplemental Experimental Procedures. Full description of all materials and methods used.

Balasooriya et al. Figure S1

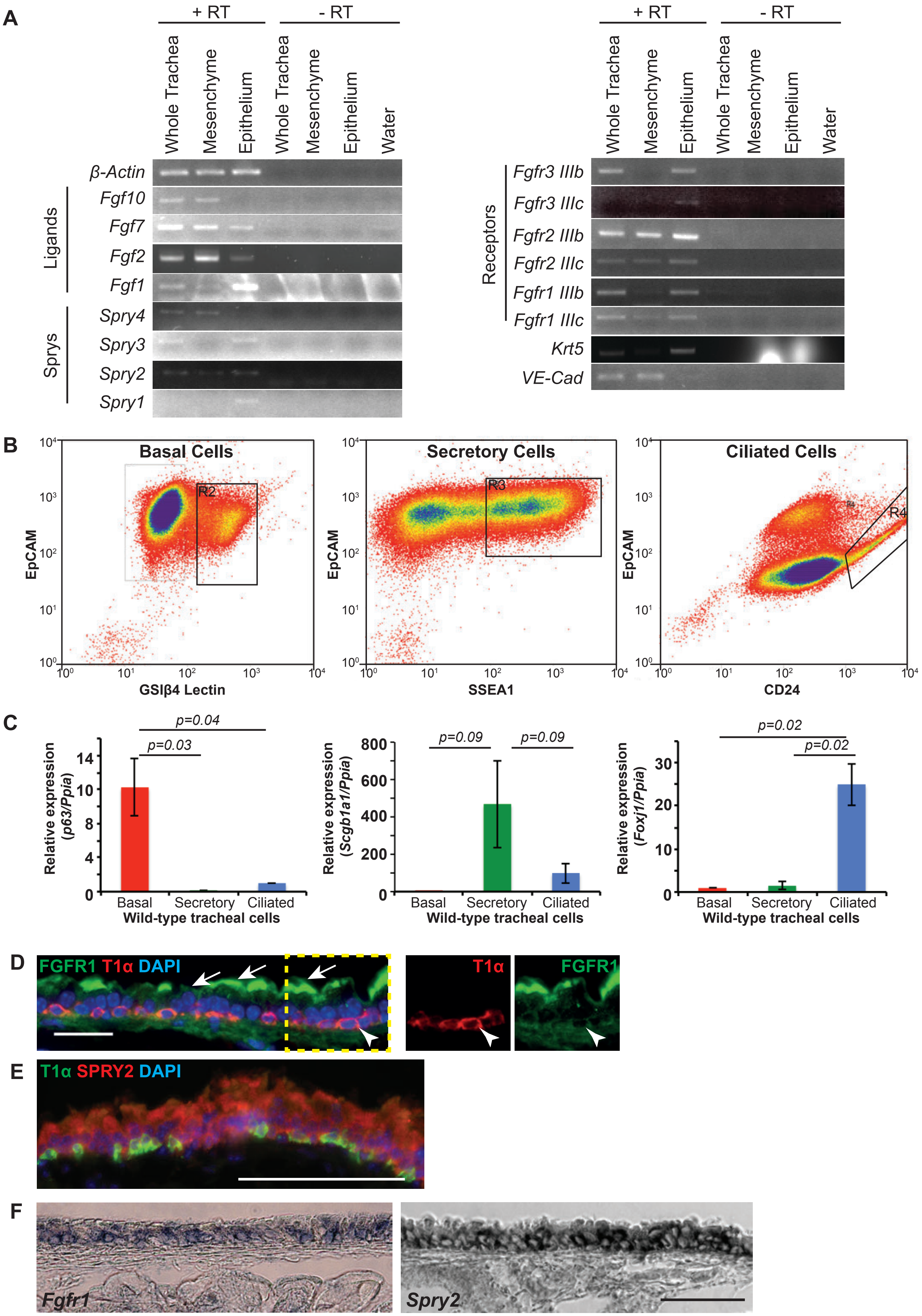

Balasooriya et al. Figure S2

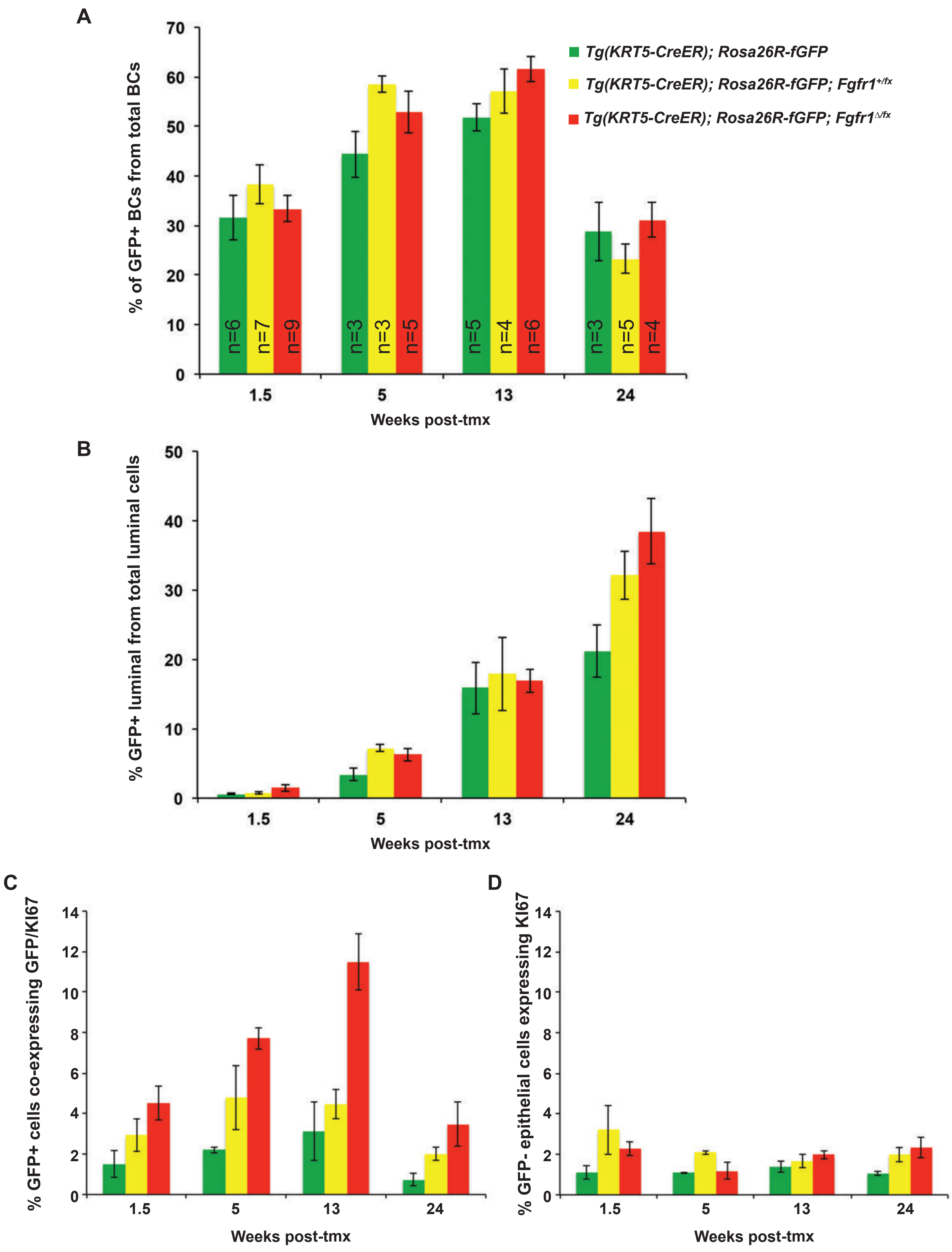

Balasooriya et al. Fig S3

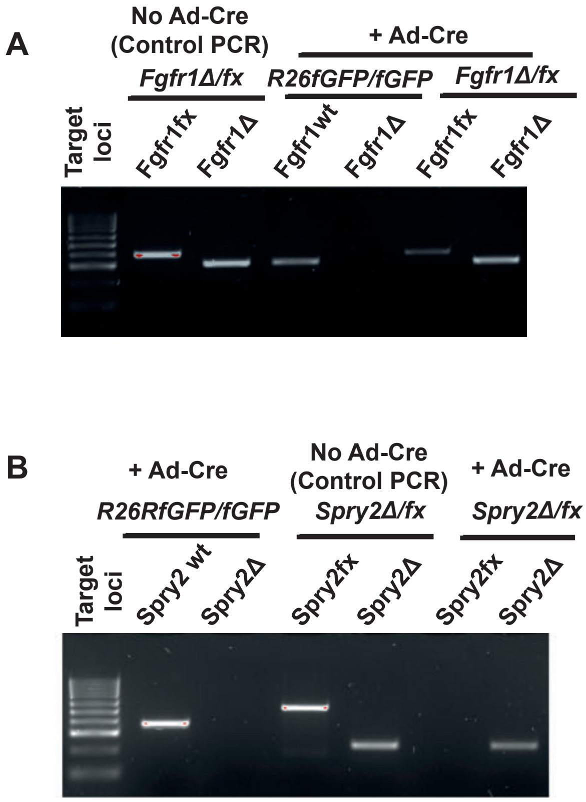

Balasooriya et al. Figure S4

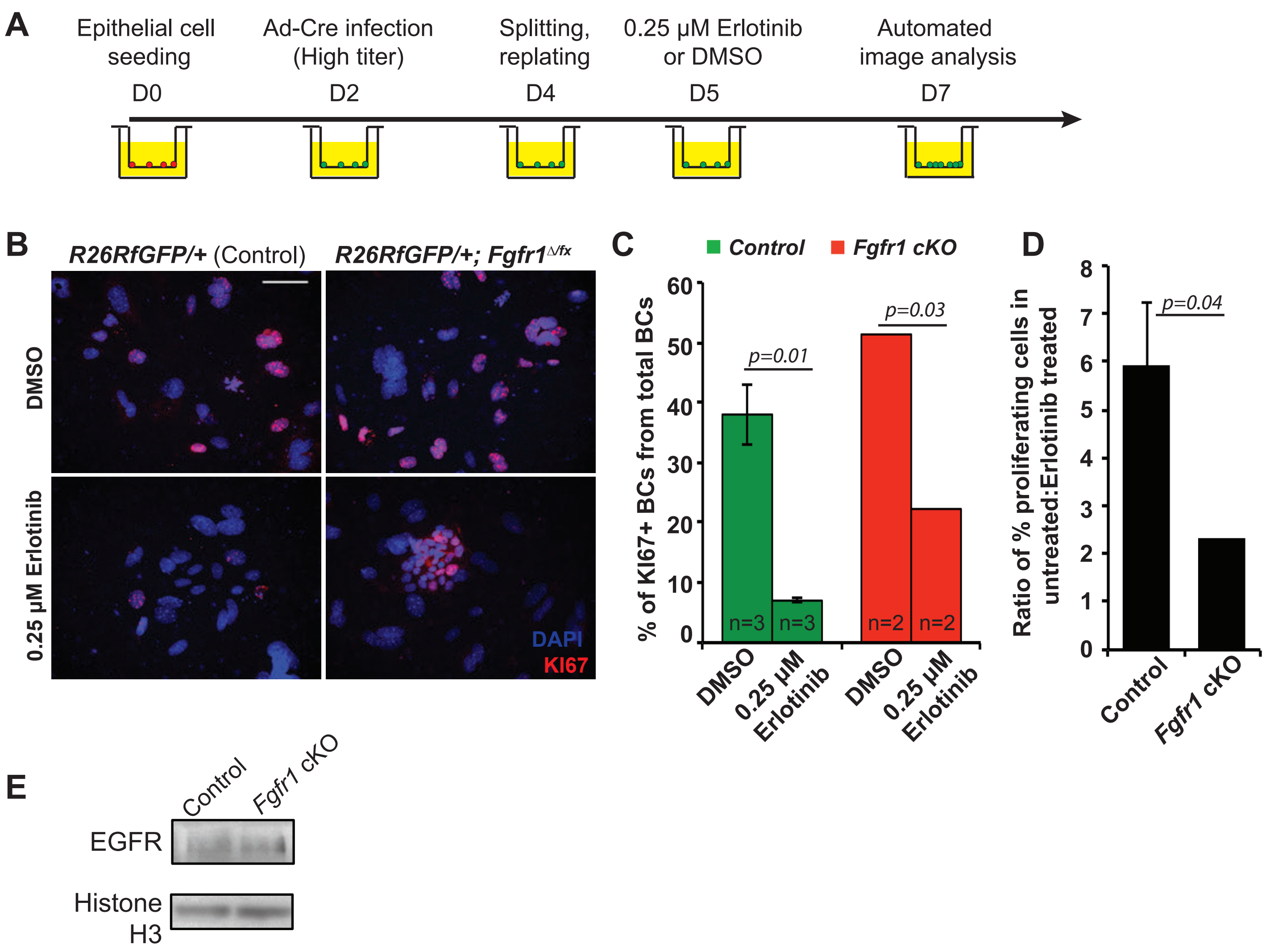

Balasooriya et al. Figure S5

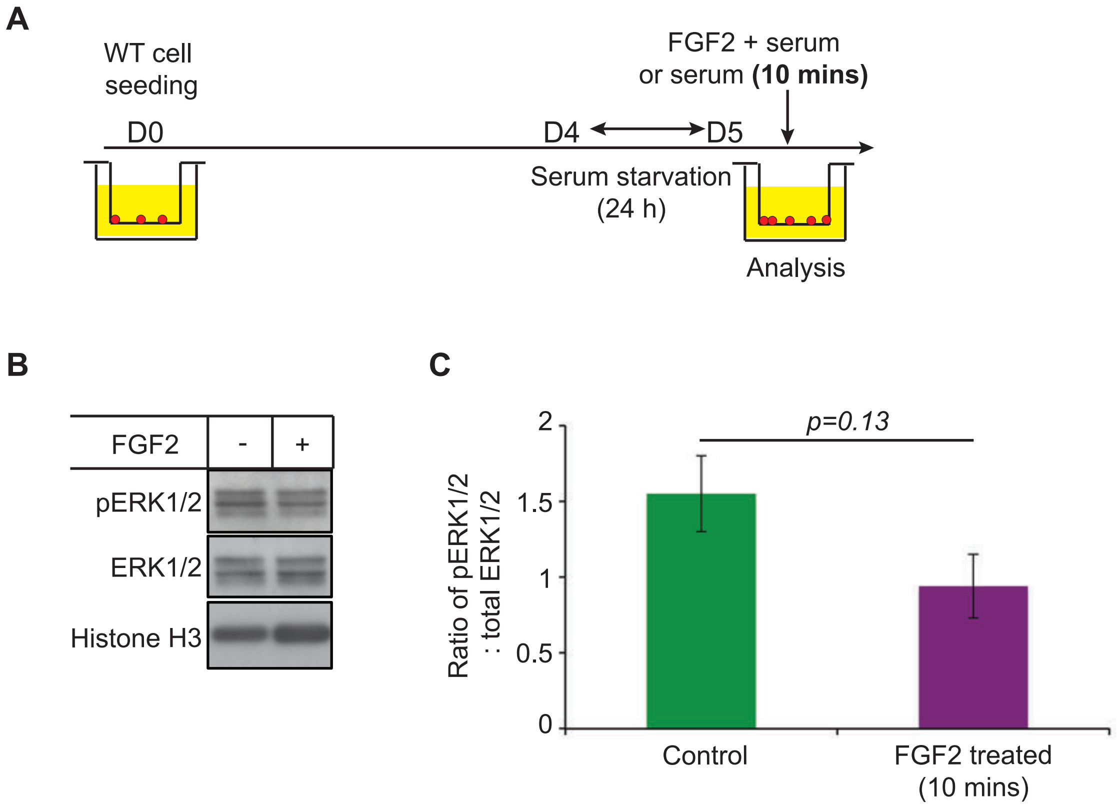

Balasooriya et al. Figure S6

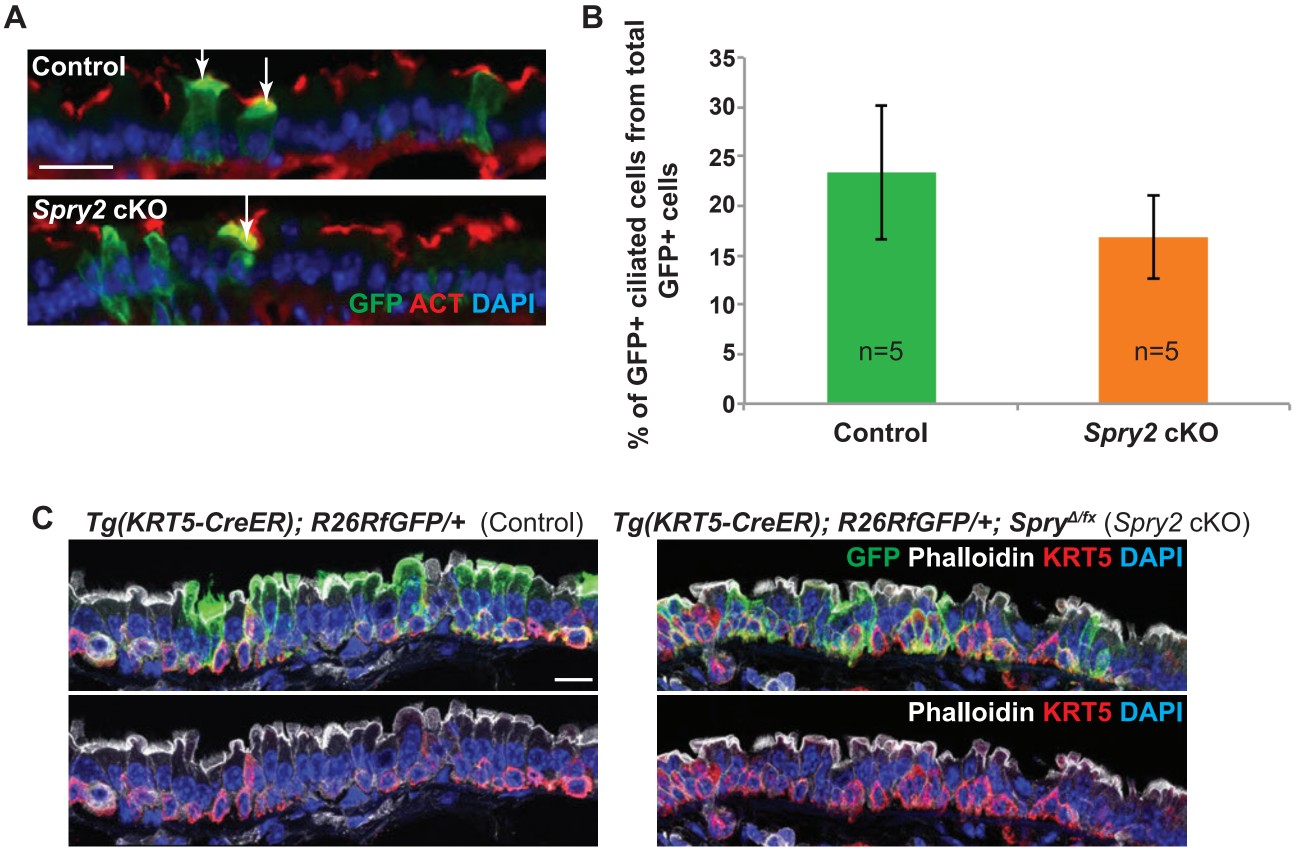

Figure S1, related to Figure 1. *Fgfr1* and *Spry2* are expressed in tracheal basal cells. (A) RT-PCR for components of the FGFR signalling pathway in the steady-state adult whole trachea, microdissected epithelium, or mesenchyme. There is widespread expression of mRNA for all receptors (except *Fgfr4* which was undetectable), all *Spry* signalling inhibitors and all tested FGF ligands. (B) Sorting of basal, secretory, and ciliated cells on surface expression of GSIβ4 lectin, SSEA1 and CD24 respectively. EpCAM is a marker of all airway epithelial cells. (C) RT-qPCR for *p63*, *Scgb1a1* and *Foxj1* in the sorted populations, normalised to *Ppia* levels, confirms basal, secretory and ciliated cell identity. Bars=SEM. (D) FGFR1 protein is expressed throughout the epithelium in wild-type tracheal epithelial sections. Green: FGFR1; red: T1α (basal cells); blue: DAPI (nuclei). FGFR1 can be seen clearly in the basal cells (arrowhead in inset) and the apical region of the luminal cells (arrows). (E) SPRY2 protein is expressed throughout the epithelium. Green: T1α (basal cells); Red: SPRY2; blue: DAPI (nuclei). (F) *Fgfr1* or *Spry2* mRNA in situ hybridisation in the steady-state adult trachea showing wide-spread epithelial expression. Scale bars = 50 μm D; 100 μm E, F.

Figure S2, related to Figures 1 and 2. *Fgfr1* heterozygous basal cells display a similar phenotype to conditional knock-out basal cells. (A-D) Graphs to show percentage of the total T1α$^+$ BCs that are also GFP$^+$ (A); percentage of the total T1α$^-$ luminal cells that are also GFP$^+$ (B); percentage of the total GFP$^+$ cells that are also KI67$^+$ (C) and percentage of the total GFP$^-$ epithelial cells that are also KI67$^+$ (D) in control, *Fgfr1* conditional heterozygous and cKO trachea. Note that there is no increase in proliferation of GFP$^-$ epithelial cells (compare D with C) suggesting that there are no widespread cell non-autonomous proliferation phenotypes in the mutant tracheae. Error bars = SEM.

Figure S3, related to Figures 3 and 6. Recombination of *Fgfr1^{fx}* and *Spry2^{fx}* in vitro. (A, B) Genomic PCR of *Fgfr1$^{\Delta/fx}$* (A) and *Spry2$^{\Delta/fx}$* (B) and control basal cells following Ad-Cre infection in vitro. Note the partial deletion of *Fgfr1^{fx}* (compare lane 1 with lane 5 in A), but the complete deletion of *Spry2^{fx}* (compare lane 3 with lane 5 in B).

Figure S4, related to Figure 3. *Fgfr1* cKO basal cells are more resistant to EGFR signalling inhibition. (A) Schematic of in vitro experiment. High titer Ad-Cre was used to delete floxed alleles, followed by passaging to provide internal controls for each biological replicate and 0.25 μM Erlotinib or DMSO treatment. Cells were scored by automated image analysis using a Fiji plug-in and thus analysis was independent of possible human bias. (B) Wild-type and *Fgfr1* cKO cells day 7 post-plating. Red: KI67 (proliferating cells); blue: DAPI (nuclei). Scale bar = 50 μm. (C) Percentage of proliferating (KI67$^+$) cells in each condition. (D) Ratio of the percentage of proliferating cells in DMSO : Erlotinib samples in control and *Fgfr1* cKO genotypes. Bars = SEM. (E) Representative western blots from control and *Fgfr1* cKO cells showing EGFR and Histone H3. No differences in EGFR levels were detectable.

Figure S5, related to Figure 3. FGF2 stimulation of wild-type basal cells results in a small drop in pERK1/2 levels. (A) Experimental time course. Wild type basal cells were serum-starved and then stimulated for 10 minutes with FGF2 and serum, or serum alone. (B) Representative western blots from control and FGF2-treated cells showing pERK1/2; total ERK1/2; Histone H3. (C) Quantification of protein in (B) normalised to loading control. Bars = SEM. There is a reproducible, but not statistically significant, decrease in the ratio of p : total ERK1/2 following FGF2 treatment.

Figure S6, related to Figure 5. Ciliated cell differentiation and epithelial organisation are normal in the *Spry2* conditional knock-outs. (A) Representative sections from control *Tg(KRT5-CreER); Rosa26R$^{fGFP/+}$* and cKO *Tg(KRT5-CreER); Rosa26R$^{fGFP/+}$; Spry2$^{\Delta/fx}$* tracheas at 13 weeks post-tmx. Green: GFP (*Rosa* reporter); red: acetylated tubulin (cilia); blue: DAPI (nuclei). Scale bar =20 μm. (B) Graph to show percentage of the total GFP$^+$ luminal cells that are also acetylated tubulin$^+$ at 13 weeks post-tmx. Bars = SEM. (C) Representative confocal z-projections of tracheal sections from control *Tg(KRT5-CreER); Rosa26R$^{fGFP/+}$* and cKO *Tg(KRT5-CreER); Rosa26R$^{fGFP/+}$; Spry2$^{\Delta/fx}$* animals at 13 weeks post-tmx. Green: GFP (*Rosa* reporter); red: KRT5 (basal cells); white: Phalloidin (F-actin); blue: DAPI (nuclei). Scale bar = 10 μm.

Supplemental Experimental Procedures

Mice

All experiments were approved by University of Cambridge local ethical review committees and conducted according to Home Office project licenses PPL80/2326 and 70/812. Mouse strains *Fgfr1*^{fx} (Xu et al., 2002), *Tg(KRT5-CreER)* (Rock et al., 2009), *Rosa26R-fGFP* (Rawlins et al., 2009), *Spry2*^{fx} (Shim et al., 2005) have all been described previously. Animals heterozygous for *Fgfr1*$^{\Delta/+}$ and *Spry2*$^{\Delta/+}$ were generated by crossing the respective floxed alleles to *Zp3-Cre* (de Vries et al., 2000). All transgenic strains were maintained on a C57Bl/6J background (at least N4 back-crosses) and a mixture of males and females were used for all experiments. No gender-specific differences were observed. All animals used for experiments were >8 weeks old. Wild-type C57Bl/6J inbred mice were used for RT-PCR, RT-qPCR, mRNA in situ hybridisation and FGF2 treatment.

Tamoxifen administration

Adult (>8 week) animals were injected intraperitoneally four times, every other day, with 0.2 mg / gram body weight tamoxifen.

Tracheal epithelial cell culture

Tracheal cells were isolated following published methods (Rock et al., 2009). Briefly, trachea were cut into small pieces and incubated in 50% Dispase II (Gibco, 16 U/ml) 20 minutes room temperature. Epithelial sheets were isolated by manual dissection and dissociated to single cells using 0.1% trypsin/EDTA. Unless otherwise stated, $5x10^4$ cells in 0.5ml MTEC/+ media (You et al., 2002) were plated on collagen-coated 12-well tissue culture inserts (BD Falcon, 353180) and grown to confluence. Differentiation was induced in confluent cultures by removal of medium from the insert and addition of MTEC serum free media to the outer chamber. Adeno-Cre (University of Iowa, Gene Transfer Vector Core) was incubated at the desired multiplicity of infection (MOI 7 for scattered recombination, MOI 2500 for complete recombination; vector pfu $1x10^6$) for 8 hours followed by washing in MTEC/+ media. Recombinant mouse FGF2 (Wellcome Trust/MRC Stem Cell Institute Tissue Culture Facility) was used at 100 ng/ml. Erlotinib (Sigma) was used at 0.25 µM. All in vitro experiments were preformed in triplicate unless otherwise stated.

Immunostaining

For protein detection tracheae were fixed in 4% paraformaldehyde at 4°C for 4 hours. Samples were washed in PBS, sucrose protected, embedded in OCT (Optimum Cutting Temperature Compound, Tissue Tek) and sectioned at 6 µm. For immunostaining of basal cell primary cultures, tissue culture inserts were washed in PBS and fixed 10 minutes in 4% paraformaldehyde at room temperature and cells were permeabilized in 0.3% triton X-100 in PBS. The following primary antibodies were used: acetylated tubulin (mouse, 1:3000, Sigma, T7451), E-cadherin (rat, 1:3000, Invitrogen, 13-1900), pERK1/2 (rabbit, 1:200, Cell signalling, 4695), FGFR1 (rabbit, 1:200, Novus Biologicals, NBP1-20067), FOXJ1 (mouse, 1:200, eBioscience, clone 2A5, 14-9965-80), GFP (chick, 1:1000, AbCam, AB13970), KRT5 (rabbit, 1:500, Covance, PRB-160P), KI67 (mouse, 1:200, BD, 550609), SCGB1A1 (goat, 1:400, Santa Cruz, sc9772), SPRY2 (rabbit, 1:200, Abcam, Ab50317), T1α (1:1000, DSHB, 8.1.1). Antigen retrieval was by boiling in 10mM sodium citrate, pH 8 for KI67, pH 6.5 for anti-pERK1/2, and pH 6 for FOXJ1. Secondary antibodies (1:2000 dilution) were Alexa Fluor conjugated from Life Technologies. DNA was stained with Dapi (Sigma) and F-actin with Phalloidin-568 (Life Technologies). Slides were mounted in Fluoromount (Sigma).

mRNA in situ hybridisation

For mRNA detection tracheas were fixed in 4% paraformaldehyde at 4°C overnight. Samples were washed in PBS, sucrose protected, embedded in OCT (Optimum Cutting Temperature Compound, Tissue Tek) and sectioned at 10 µm. DIG-labelled anti-sense mRNA probes to *Fgfr1* (EST ID: AW495528) or *Spry2* (Minowada et al., 1999) were prepared using standard methods (DIG RNA labelling Kit, Roche, 11175025910) and hybridised to tissue sections overnight at 65°C in a buffer containing 50% formamide. Following stringent post-hybridisation washes in 0.2x SSC, slides were blocked and incubated with Alkaline Phosphatase (AP) coupled anti-DIG (1:2000 – Roche 12930023). AP activity was visualized using BCIP (Roche, 1383213)/ NBT (Roche, 1383213). Sections were mounted in 70% glycerol and imaged in a Zeiss AxioImager compound microscope.

Microscopy and Image scoring

Slides were imaged on a Zeiss AxioImager compound microscope, or either an Olympus FV1000 or Leica Sp8/Sp5 confocal microscope where stated. Most experiments were scored manually in Fiji (ImageJ). For cryosections every epithelial cell along the entire proximal to distal length of a longitudinal section from the centre of the trachea was scored. Basal cells were scored as either T1α$^+$ or KRT5$^+$ with an apical surface which

did not extend to the lumen. Scoring of other cell types was based on markers as described in the text. For cultured cells, at least three random fields of view from each insert were scored. To assess cell density in control and *Spry2* cKO tracheas, straight lines were drawn along the basement membrane in regions with large patches of GFP$^+$ cells using Fiji and the number of Dapi$^+$ nuclei along the line was scored. For the Erlotinib experiments an automated scoring system was employed. Cells were segmented on the basis of DAPI$^+$ nuclei and the KI67 fluorescence intensity was measured using the Gurdon Institute Imaging Facility's Fiji plugin, ObjectScan. Cells were scored as KI67$^+$ by the plug-in if their fluorescence intensity exceeded a defined threshold.

RT-PCR

Samples were enriched for epithelium or mesenchyme using Dispase digestion and manual peeling as described for tracheal epithelial cell culture. Total RNA was extracted using Trizol reagent and cDNA was synthesised using Superscript III reverse transcriptase (Life Technologies). Primer sequences:
Fgf1-F: TTGACCAATGCTGAGCCTAC
Fgf1-R: AGAGCAGTTTGGGCTTTTTG
Fgf2-F: CAACCGGTACCTTGCTATGA
Fgf2-R: ACTGCCCAGTTCGTTTCAGT
Fgf7-F: CCATGAACAAGGAAGGGAAA
Fgf7-R: TTGACAGGAATCCCCTTTTG
Fgf10-F: CCATGAACAAGAAGGGGAAA
Fgf10-R: CTCTCCTGGGAGCTCCTTTT
Fgfr1 IIIb-F: CCAACCTCTAACCGCAGAAC
Fgfr1 IIIb-R: TCGTCGTCGTCATCATCTTC
Fgfr1 IIIc-F: TCACAGCCACTCTCTGCACT
Fgfr1 IIIc-R: CCAGTTGATGCTCTGCACAT
Fgfr2 IIIb-F: ACCGAGAAGATGCATGGAGAAGC
Fgfr2 IIIb-R: TAAGGCTCCAGTGCTGGTTT
Fgfr2 IIIc-F: GCTAGGACGGTAGACA
Fgfr2 IIIc-R: TCTGGTTGCTCCTGTTCTCA
Fgfr3 IIIb-F: TGAAGCACGTGGAAGTGAAC
Fgfr3 IIIb-R: GGTGGCTCGACAGAGGTACT
Fgfr3 IIIc-F: TGAAGCACGTGGAAGTGAAC
Fgfr3 IIIc-R: TCTAGCTCCTTGTCGGTGGT
Fgfr4-F: GGAGGAGCTCTTCTCACTGC
Fgfr4-R: CAGGACCTTGTCCAGAGCTT
Spry1-F: GGCCTATTAGGACGGTCTCC
Spry1-R: CAGTGGGACTGTGAACAGGA
Spry2-F: ACATCGCTGGAAGAAGAGGA
Spry2-R: CAGGTCTTGGCAGTGTGTTC
Spry3-F: TTCTGCATGGTCATCATTTG
Spry3-R: GGCACTTGCGGTGATTTA
Spry4-F: GGTTCGGGGATTTACACAGA
Spry4-R: CATGACTGAGCTGGGATTCA
K5-F: TCCAGAACGCCATTGCTGAAG
K5-R: CCGTAGCCAGAAGAGACACTGTTTG
VE-Cadherin-F: AAGCAGAACCTGACCTGGAA
VE-Cadherin-R:GTGAGGGGCA ATGACAATCT
β-Actin-F: CCCTGAAGTACCCCATTGAA
β-Actin-F: CTTTTCACGGTTGGCCTTAG.

RT-qPCR

Primary tracheal epithelial cells were isolated as for cell culture. Cells were resuspended in FACS buffer (2.0% FBS in PBS). Cells were stained with EpCAM-PECy7 (1:100; 25-5791-80, eBiosciences), GSIβ4-FITC (1:100; L2895, Sigma), SSEA1-Alexa Fluor® 647 (1:50; 125608, BioLegend), and CD24-PE (1:75; 553262, BD Pharmingen) for 30 minutes on ice (Zhao et al., 2014). Cells were sorted using a fluorescence-activated cell sorting MoFlo flow cytometer. Wild-type basal cells were sorted as EpCAM$^+$, GSIβ4 lectin$^+$ positive; secretory cells as EpCAM$^+$, SSEA1$^+$; ciliated cells as EpCAM$^+$, CD24$^+$. GFP$^+$ basal cells from control and *Fgfr1* cKO tracheae were sorted using endogenous GFP fluorescence from *Rosa26R-fGFP* and GSIβ4-647 (1:100, 132450, Life Technologies). Total RNA was extracted using Qiagen RNEasy Mini Kit and cDNA was synthesised using Superscript III reverse transcriptase (Life Technologies). Taqman gene expression assays (Life Technologies) were used for expression analysis: *Fgfr1* (Mm00438930_m1); *Foxj1* (Mm01267279_m1); *p63*

(Mm00495791_m1); *Ppia* (Mm02342429_g1); *Scgb1a1* (Mm00442046_m1); *Spry2* (Mm00442344_m1).

Immunoblot analysis

Cultured cells were collected in Cell Extraction Buffer (Invitrogen, FNN0011) with 1x cocktail protease inhibitor (Roche 04693116001) and PMSF (Sigma, P7626). Protein concentration of the lysate was measured by Nanodrop and proteins separated on 10 or 12% SDS-PAGE gels before transfer onto Millipore Immobilon-P PVDF Membrane (Merck Millipore, IPVH00010). Membranes were blocked with 5% dried skim milk and probed with p-AKT(S473) (Cell Signalling, 3787, 1:3000), pan-AKT (Cell Signalling, 4691, 1:1000), dpERK1/2 (Cell Signalling, 4370, 1:300), ErRK1/2 (Cell Signalling, 4695, 1: 300), SPRY2 (Abcam, ab50317, 1:300), SOX2 (Abcam, Cat: ab97959, 1:3000), H3 (Abcam, ab39655, 1:10000), EGFR (Millipore, 04-290, 1:300) primary antibodies. Detection was with HRP-conjugated secondaries goat anti-rabbit IgG H&L (Abcam, ab97051, 1:10000), goat anti-mouse IgG H&L (Abcam, ab97023, 1:10000) and enhanced chemiluminescense (Thermo Scientific, PI-32109). Representative blots are shown. Quantitation is based on the analysis of protein from three biological replicates which were separated on the same polyacrylamide gel. Band intensity was analysed in Fiji normalised to the loading control.

Statistics

All p-values shown were obtained using an unpaired two-tailed student's t-test with unequal variance.

REFERENCES

de Vries, W.N., Binns, L.T., Fancher, K.S., Dean, J., Moore, R., Kemler, R., and Knowles, B.B. (2000). Expression of Cre recombinase in mouse oocytes: a means to study maternal effect genes. Genesis *26*, 110-112.

Minowada, G., Jarvis, L.A., Chi, C.L., Neubuser, A., Sun, X., Hacohen, N., Krasnow, M.A., and Martin, G.R. (1999). Vertebrate Sprouty genes are induced by FGF signaling and can cause chondrodysplasia when overexpressed. Development *126*, 4465-4475.

Rawlins, E.L., Okubo, T., Xue, Y., Brass, D.M., Auten, R.L., Hasegawa, H., Wang, F., and Hogan, B.L. (2009). The role of Scgb1a1+ Clara cells in the long-term maintenance and repair of lung airway, but not alveolar, epithelium. Cell stem cell *4*, 525-534.

Rock, J.R., Onaitis, M.W., Rawlins, E.L., Lu, Y., Clark, C.P., Xue, Y., Randell, S.H., and Hogan, B.L. (2009). Basal cells as stem cells of the mouse trachea and human airway epithelium. Proc Natl Acad Sci U S A *106*, 12771-12775.

Shim, K., Minowada, G., Coling, D.E., and Martin, G.R. (2005). Sprouty2, a mouse deafness gene, regulates cell fate decisions in the auditory sensory epithelium by antagonizing FGF signaling. Dev Cell *8*, 553-564.

Xu, X., Qiao, W., Li, C., and Deng, C.X. (2002). Generation of Fgfr1 conditional knockout mice. Genesis *32*, 85-86.

You, Y., Richer, E.J., Huang, T., and Brody, S.L. (2002). Growth and differentiation of mouse tracheal epithelial cells: selection of a proliferative population. Am J Physiol Lung Cell Mol Physiol *283*, L1315-1321.

Zhao, R., Fallon, T.R., Saladi, S.V., Pardo-Saganta, A., Villoria, J., Mou, H., Vinarsky, V., Gonzalez-Celeiro, M., Nunna, N., Hariri, L.P*., et al.* (2014). Yap Tunes Airway Epithelial Size and Architecture by Regulating the Identity, Maintenance, and Self-Renewal of Stem Cells. Dev Cell *30*, 151-165.
