# Supplementary material for: An FGFR1-SPRY2 Signaling Axis Limits Basal Cell Proliferation in the Steady-State Airway Epithelium
Source: Dev Cell. 2016 Apr 4;37(1):85–97. doi: 10.1016/j.devcel.2016.03.001 (PMC4825408; doi:10.1016/j.devcel.2016.03.001)
Supplement: Document S2. Article plus Supplemental Information [file mmc2.pdf]

Article

Developmental Cell

An FGFR1-SPRY2 Signaling Axis Limits Basal Cell Proliferation in the Steady-State Airway Epithelium

Graphical Abstract

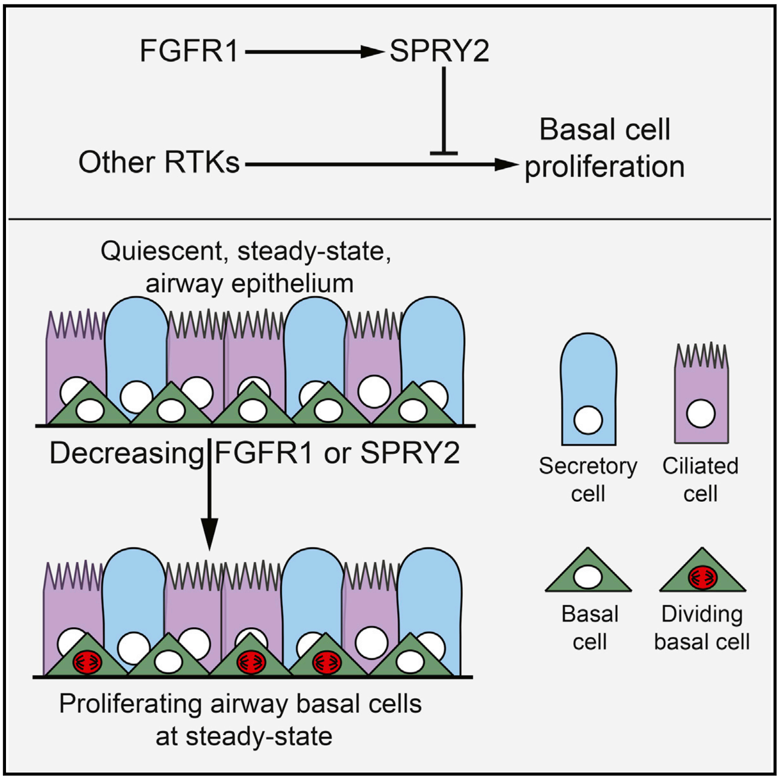

Highlights

- FGFR1 signaling is important for inhibiting airway stem cell proliferation
- FGFR1 post-translationally activates SPRY2, which inhibits activity of other RTKs
- Balancing the activity of different RTKs tunes airway stem cell proliferation rates
- Ciliated cell fate specification also requires FGFR1 but does not require SPRY2

Authors

Gayan I. Balasooriya, Jo-Anne Johnson, M. Albert Basson, Emma L. Rawlins

Correspondence

e.rawlins@gurdon.cam.ac.uk

In Brief

Airway basal cells are highly quiescent at homeostasis. Rawlins et al. show that this quiescence is actively maintained, in part by an FGFR1-SPRY2 signaling mechanism. This FGFR1 signaling limits steady-state basal cell proliferation by repressing an RTK-mediated pro-proliferative signal.

Balasooriya et al., 2016, Developmental Cell *37*, 85–97
April 4, 2016 ©2016 The Authors
http://dx.doi.org/10.1016/j.devcel.2016.03.001

CellPress

An FGFR1-SPRY2 Signaling Axis Limits Basal Cell Proliferation in the Steady-State Airway Epithelium

Gayan I. Balasooriya,[1] Jo-Anne Johnson,[1] M. Albert Basson,[2] and Emma L. Rawlins[1,*]
[1]Wellcome Trust/CRUK Gurdon Institute, Wellcome Trust/MRC Stem Cell Institute, Department of Pathology, University of Cambridge, Cambridge CB2 1QN, UK
[2]Department of Craniofacial Development and Stem Cell Biology, King's College London, London SE1 9RT, UK
*Correspondence: e.rawlins@gurdon.cam.ac.uk
http://dx.doi.org/10.1016/j.devcel.2016.03.001

SUMMARY

The steady-state airway epithelium has a low rate of stem cell turnover but can nevertheless mount a rapid proliferative response following injury. This suggests a mechanism to restrain proliferation at steady state. One such mechanism has been identified in skeletal muscle in which pro-proliferative FGFR1 signaling is antagonized by SPRY1 to maintain satellite cell quiescence. Surprisingly, we found that deletion of *Fgfr1* or *Spry2* in basal cells of the adult mouse trachea caused an increase in steady-state proliferation. We show that in airway basal cells, SPRY2 is post-translationally modified in response to FGFR1 signaling. This allows SPRY2 to inhibit intracellular signaling downstream of other receptor tyrosine kinases and restrain basal cell proliferation. An FGFR1-SPRY2 signaling axis has previously been characterized in cell lines in vitro. We now demonstrate an in vivo biological function of this interaction and thus identify an active signaling mechanism that maintains quiescence in the airway epithelium.

INTRODUCTION

The airway epithelium of both mouse and humans is essentially quiescent at steady state, with an extremely low rate of stem cell proliferation (Cole et al., 2010; Kauffman, 1980; Teixeira et al., 2013). Nevertheless, airway basal cells (BCs) can rapidly enter the cell cycle in response to luminal cell loss (Hong et al., 2004; Pardo-Saganta et al., 2015; Rawlins et al., 2007). Several paracrine signaling pathways that promote airway stem cell proliferation following injury have been characterized (reviewed in Hogan et al., 2014). In addition, autocrine signaling mechanisms can initiate airway proliferation in response to local damage (Vermeer et al., 2003). A critical question remains: are there are also mechanisms which actively inhibit airway proliferation at homeostasis and therefore function to maintain quiescence?

In overall organization the mouse trachea is very similar to human smaller airways (Hackett et al., 2011; Rock et al., 2010; Teixeira et al., 2013). The adult mouse tracheal epithelium comprises three main cell types. BCs include both slowly dividing stem cells and committed luminal precursors (Mori et al., 2015; Rock et al., 2009; Watson et al., 2015). Luminal secretory cells can self-renew and produce luminal ciliated cells, while ciliated cells are terminally differentiated (Rawlins and Hogan, 2008; Rawlins et al., 2007, 2009). In vitro and in vivo evidence suggests that airway BC proliferation requires epidermal growth factor receptor (EGFR) activity (Brechbuhl et al., 2014; You et al., 2002). Moreover, inhibition of EGFR signaling via contact inhibition is necessary to restrain BC proliferation following injury (Lu et al., 2013). WNT and Notch signaling can also promote BC proliferation in some contexts (Giangreco et al., 2012; Paul et al., 2014; Rock et al., 2011). By contrast, YAP prevents differentiation of BCs (Mahoney et al., 2014; Zhao et al., 2014). However, no specific signaling pathways that actively inhibit BC proliferation at steady state have been identified.

In other organs, stem cell quiescence is actively maintained by feedback inhibition. For example, in the satellite cells of skeletal muscle steady-state quiescence requires the function of specific receptor tyrosine kinase (RTK) inhibitors, SPRY proteins, to antagonize pro-proliferative fibroblast growth factor receptor 1 (FGFR1) signaling (Chakkalakal et al., 2012; Shea et al., 2010). We speculated that similar mechanisms would operate in the steady-state airway epithelium.

FGFR signaling has been extensively studied in lung development and the smaller conducting airways (e.g., Abler et al., 2009; Volckaert et al., 2011, 2013; Yin et al., 2011) where, similar to its role in muscle, it has been found to have a pro-proliferative function. However, the role of FGFR signaling in airway BCs remains undetermined. We therefore tested whether antagonism of FGFR1 activity by SPRY proteins is required for BC quiescence. Surprisingly, we found that deletion of either *Fgfr1* or *Spry2* resulted in increased levels of BC proliferation. We demonstrate that in airway BCs, SPRY2 is post-translationally modified downstream of FGFR1, allowing SPRY2 to antagonize signaling from other RTKs, most likely EGFR, and maintain quiescence. There is a well-documented in vitro relationship between FGFR1-mediated modification of SPRY2 and RAS-ERK inhibition (Lao et al., 2006, 2007). However, a role for this interaction has never previously been identified in vivo.

CrossMark

This is an open access article under the CC BY license (http://creativecommons.org/licenses/by/4.0/).

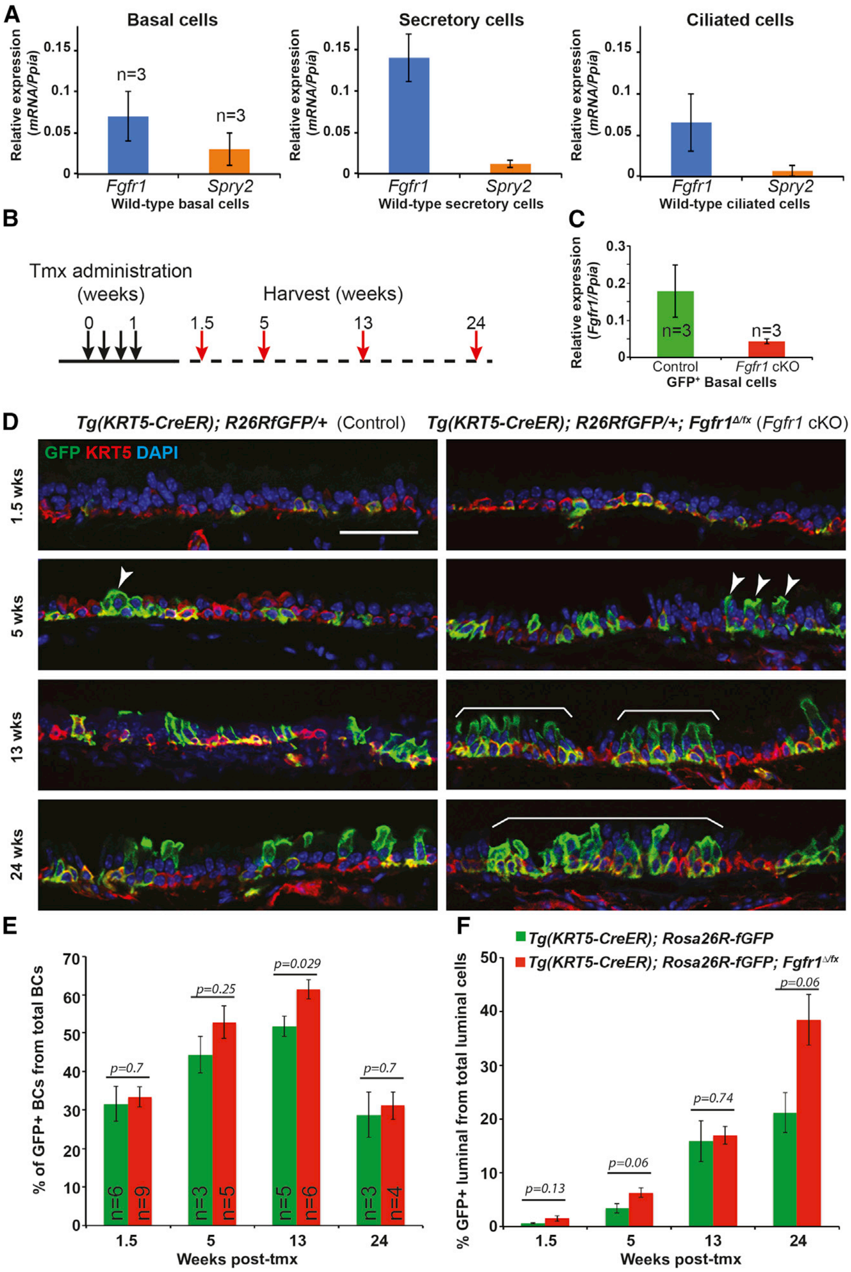

Figure 1. Deletion of *Fgfr1* in BCs Results in Altered Tracheal Epithelial Homeostasis

(A) Relative expression of *Fgfr1* and *Spry2* mRNA in sorted wild-type basal, secretory, and ciliated cells.

(B) Schematic of *Fgfr1* conditional knockout experiment.

(C) Relative expression of *Fgfr1* mRNA in sorted GFP^{+} BCs from control and *Fgfr1* cKO mice 3 weeks after tmx administration.

(D) Representative confocal sections from control *Tg(KRT5-CreER)*; *Rosa26R$^{fGFP/+}$* and cKO *Tg(KRT5-CreER)*; *Rosa26R$^{fGFP/+}$*; *Fgfr1$^{\Delta/fx}$* tracheae. Green, GFP (reporter); red, KRT5 (BCs); blue, DAPI (nuclei). Increased labeling of luminal cells is visible in the *Fgfr1* cKOs by 5 weeks (arrowheads), becoming large patches of labeled cells (brackets) by 13 and 24 weeks.

(E) Percentage of the total T1α^{+} BCs that are also GFP^{+}.

(F) Percentage of total T1α^{-} luminal cells that are also GFP^{+}.

Error bars denote SEM. Scale bars, 50 μm. See also Figures S1 and S2.

RESULTS

FGFR1 Signaling Is Required for Normal Tracheal Cellular Homeostasis

FGFR signaling pathway components are readily detected in the steady-state adult mouse trachea by RT-PCR (Figure S1A). *Fgfr1* and *Spry2* mRNA were also detected in purified BC, secretory, and ciliated cell populations by qRT-PCR (Figures 1A, S1B, and S1C) and by single-cell qRT-PCR (Watson et al., 2015). Moreover, FGFR1 protein and mRNA were detected in BCs and luminal cells in the intact mouse trachea (Figures S1D and S1F). We conditionally deleted *Fgfr1* and activated a GFP reporter in tracheal BCs using *Tg(KRT5-CreER)*; *Rosa26R-fGFP*; *Fgfr1$^{\Delta/fx}$* (*Fgfr1* conditional knockout, cKO) and control *Tg(KRT5-CreER)*; *Rosa26R-fGFP* mice. Four doses of tamoxifen (tmx) were administered to adult (>8 weeks old) mice (Figure 1B). To confirm deletion of *Fgfr1^{fx}* in the GFP^{+} BCs, we performed qRT-PCR on pools of isolated GFP^{+} BCs and showed that the level of *Fgfr1* mRNA in the *Fgfr1* cKOs was reduced to 20% of the control level (Figure 1C). This result showed that widespread co-recombination of the *Fgfr1^{fx}* and *Rosa26R^{fGFP}* loci was occurring, and we used GFP^{+} cells as a surrogate marker for *Fgfr1$^{\Delta/\Delta}$* cells in further experiments. However, it is important to note that *Rosa* and *Fgfr1* co-recombination is ~80%, and we therefore underestimate the extent of the *Fgfr1* phenotype using this scoring method.

Tracheae were harvested at intervals and processed for histology to assess the contribution of GFP^{+}, *Fgfr1$^{\Delta/\Delta}$* BCs to the epithelium during normal homeostatic turnover. At 1.5 weeks after tmx administration, ~30% of total BCs were GFP^{+} in both *Fgfr1* cKOs and controls. In control animals this percentage increased modestly to 40%–50% at 5–13 weeks after tmx administration, before dropping to initial levels by 24 weeks. This change was much more marked in the *Fgfr1* cKO mice, in which the percentage of labeled BCs peaked at 60% at 13 weeks before returning to initial levels (Figures 1D and 1E). In both genotypes, labeled BCs produced labeled luminal cells as expected. However, luminal cell production was greater in the *Fgfr1* cKO mice, with levels of GFP^{+} luminal cells reaching 38% ± 2% of the total luminal cell population by 24 weeks, compared with 21% ± 4% in the controls (Figure 1F). *Tg(KRT5-CreER)*; *Rosa26R-fGFP*; *Fgfr1$^{+/fx}$* (*Fgfr1* conditional heterozygous) mice displayed a phenotype similar to that

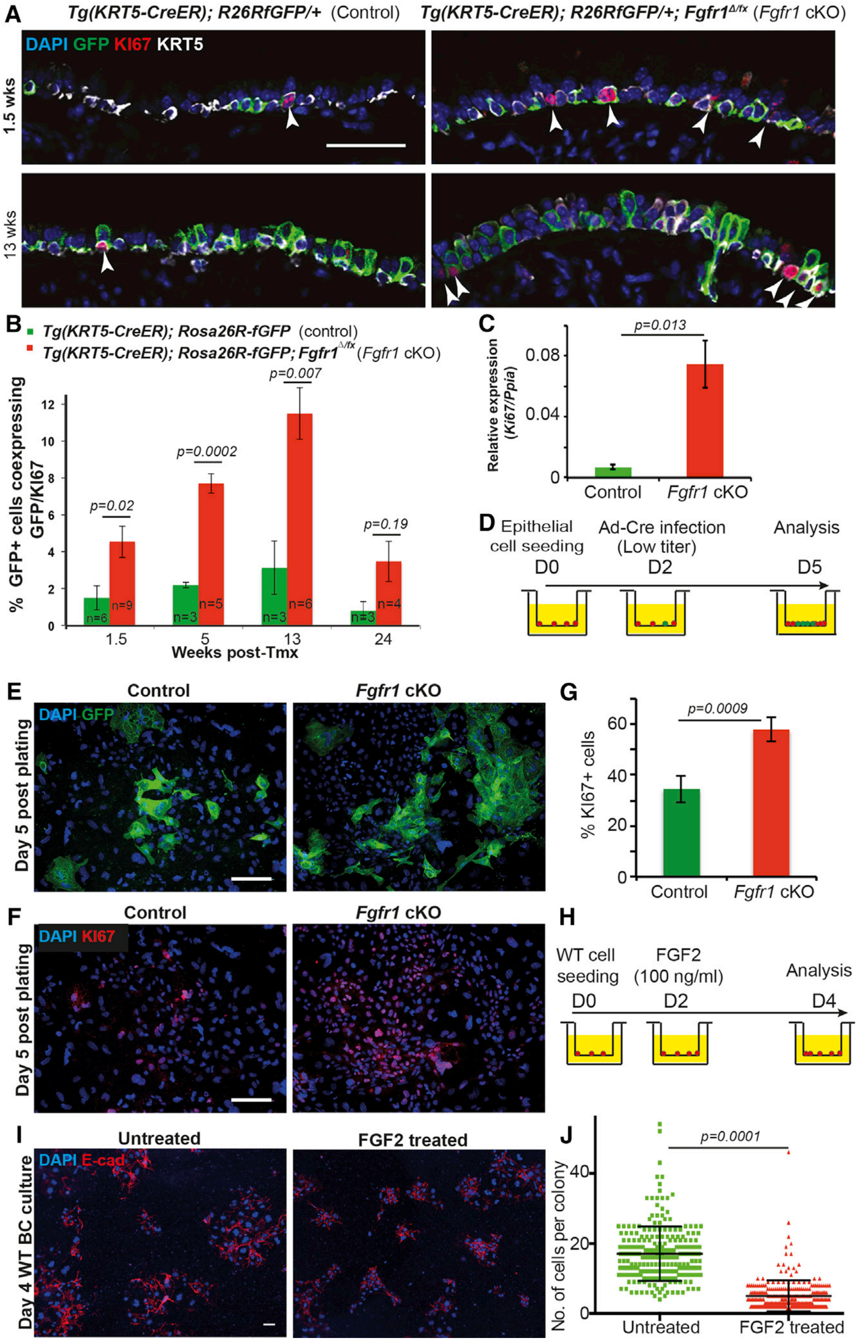

Figure 2. Loss of FGFR1 Signaling Results in Increased Levels of BC Proliferation

(A) Representative confocal sections from control *Tg(KRT5-CreER)*; *Rosa26R$^{fGFP/+}$* and cKO *Tg(KRT5-CreER)*; *Rosa26R$^{fGFP/+}$*; *Fgfr1$^{\Delta/fx}$* tracheae at 1.5 and 13 weeks after tmx administration. Green, GFP (reporter); red, KI67 (proliferating cells); white, KRT5 (BCs); blue, DAPI (nuclei). Arrowheads indicate KI67^{+} cells.

(B) Percentage of GFP^{+} cells that co-express KI67.

(C) Relative expression of *Ki67* mRNA in sorted GFP^{+} BCs from control and *Fgfr1* cKO mice 3 weeks after tmx administration.

(D) Schematic of in vitro experiment.

(E) Low-titer Ad-Cre infection induces sporadic recombination in control *Rosa26R$^{fGFP/+}$* and cKO *Rosa26R$^{fGFP/+}$*; *Fgfr1$^{\Delta/fx}$* BCs. Green, GFP (reporter); blue, DAPI (nuclei).

(F) *Fgfr1* deletion in vitro results in increased BC proliferation. Red, KI67 (proliferating cells); blue, DAPI (nuclei).

(G) Percentage of proliferating (KI67^{+}) cells in vitro.

(H) Schematic for in vitro experiment. Wild-type (WT) BCs plated at low density (3 × 10^4 cells/insert) and exposed to 100 ng/ml FGF2 from day 2.

(I) FGF2-treated and control wild-type BCs at day 4 after plating. Red, E-cadherin (lateral cell membrane); blue, DAPI (nuclei).

(J) Number of DAPI^{+} cells per colony in control and FGF2-treated cells.

Error bars denote SEM, Scale bars represent 50 μm in (A), (E), and (F), and 20 μm in (I). See also Figure S2.

of the cKOs (Figures S2A and S2B). Thus, greater numbers of both basal and luminal cells are produced by the *Fgfr1$^{\Delta/\Delta}$* BCs than controls, showing that FGFR1 signaling influences the rate of epithelial cell turnover in the steady-state trachea.

Increased Levels of Basal Cell Proliferation in the *Fgfr1* cKO Tracheae

We assessed proliferation rates using KI67 antibody staining. This showed that in vivo rates of proliferation were >3-fold higher in the *Fgfr1$^{\Delta/\Delta}$* BCs than in controls (Figures 2A and 2B). Moreover, the level of *Ki67* mRNA in GFP^{+} BCs isolated from *Fgfr1* cKO tracheae was increased 11-fold compared with controls (Figure 2C). Proliferation levels were intermediate in the *Fgfr1* conditional heterozygotes (Figure S2C). We confirmed the increase in BC proliferation in vitro using a limiting dose of Cre recombinase adenovirus (Ad-Cre) to induce recombination in small numbers of cells from *Rosa26R-fGFP*; *Fgfr1$^{\Delta/fx}$* and control *Rosa26R-fGFP* tracheae (Figure 2D). The *Fgfr1$^{\Delta/\Delta}$* cells produced larger GFP^{+} colonies than the controls, and these cultures also contained higher numbers of KI67^{+} cells (Figures 2E–2G).

We predicted that if deletion of *Fgfr1* results in BC proliferation, activating FGFR1 signaling in vitro would inhibit growth of BC colonies. FGF2 is expressed in the homeostatic trachea (Figure S1A), is known to activate FGFR1 preferentially in vitro, and is strongly linked to FGFR1 activation in multiple in vivo situations (Arai et al., 2015; Chakkalakal et al., 2012; Garmy-Susini et al., 2004; Hackett et al., 2011; Hadjab et al., 2013; Kranenburg et al., 2005; Ornitz et al., 1996). We therefore tested the effects of the addition of exogenous FGF2 protein on the proliferation of wild-type primary BCs in culture. We plated wild-type BCs at low density and added FGF2 on culture day 2 after colonies had become established (Figure 2H). As predicted, addition of FGF2 had the

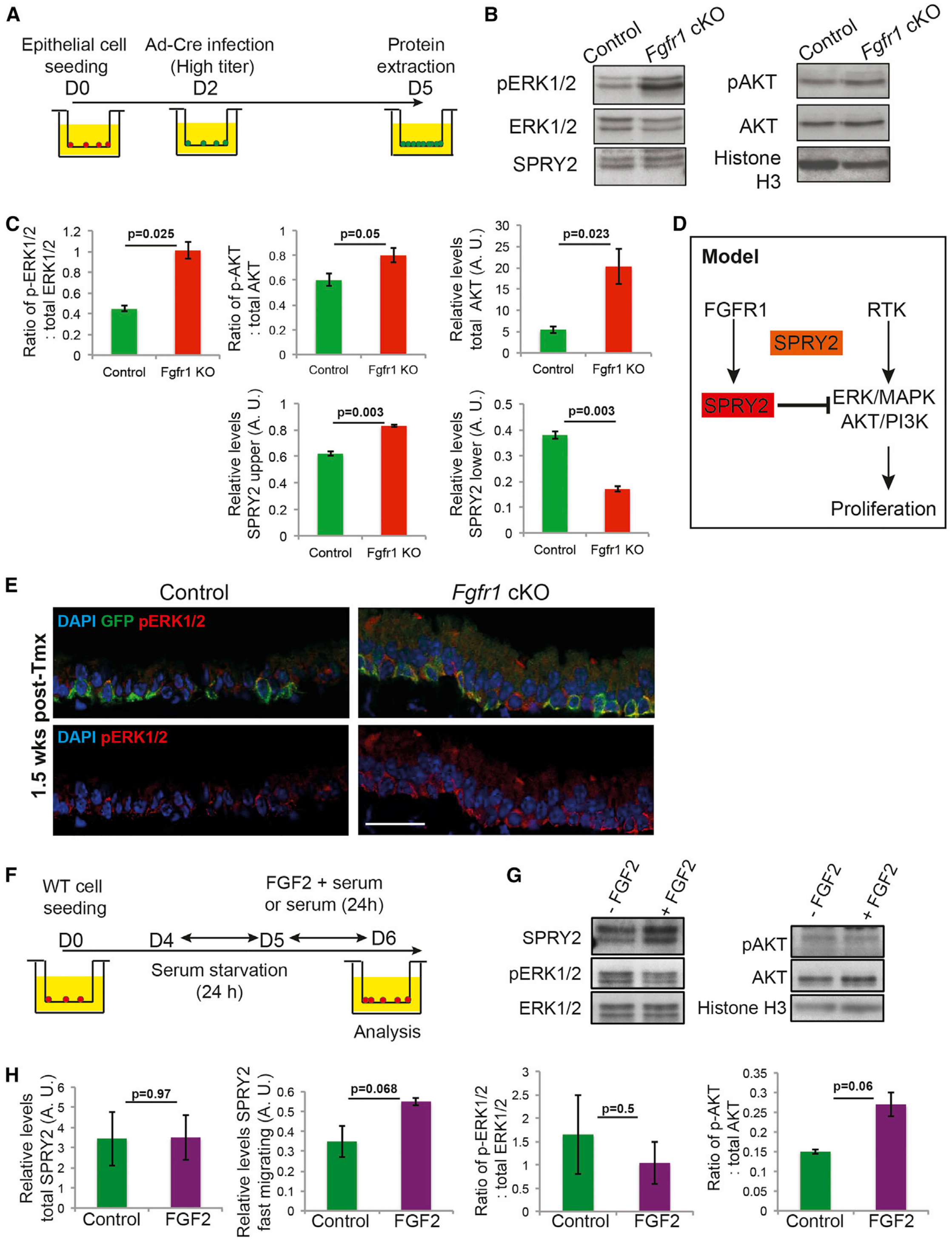

Figure 3. Loss of FGFR1 Signaling in BCs Leads to an Increase in Phosphorylation of Downstream Effector Proteins and a Decrease in Levels of a SPRY2 Isoform

(A) Schematic of in vitro experiment.

(B) Representative western blots from control and *Fgfr1* cKO day-5 BCs showing pERK1/2, total ERK1/2, pAKT, total AKT, SPRY2, and histone H3.

(C) Quantification of protein in (B).

(D) Model. A major function of FGFR1 in BCs is to post-translationally modify SPRY2, resulting in a SPRY2 isoform which can negatively regulate the ERK/MAPK and AKT/PI3K pathways downstream of other RTKs.

(E) Confocal images of control *Tg(KRT5-CreER)*; *Rosa26R$^{fGFP/+}$* and cKO *Tg(KRT5-CreER)*; *Rosa26R$^{fGFP/+}$*; *Fgfr1$^{\Delta/fx}$* tracheal sections at 1.5 weeks after tmx administration showing an increase in pERK1/2 levels in the *Fgfr1* cKO BCs. Green, GFP (reporter); red, pERK1/2 (active ERK1/2); blue, DAPI (nuclei). Scale bar, 25 μm.

(legend continued on next page)

opposite effect to removal of *Fgfr1* and significantly reduced colony size (Figures 2I and 2J).

Fgfr1$^{\Delta/\Delta}$ BCs Have Increased Levels of MAPK and AKT Pathway Activity

To identify the mechanism by which FGFR1 influences BC proliferation, we assessed the levels of activity of well-characterized downstream intracellular signaling pathways. Given that we had successfully replicated the proliferation phenotype in primary cell culture (Figures 2D–2F), we used primary cultures of *Fgfr1*$^{\Delta/fx}$ cells with a high dose of Ad-Cre and harvested cells 3 days after infection for western analysis of the whole cell lysate (Figure 3A). Genotyping of the cultures suggested that many cells were *Fgfr1*$^{\Delta/\Delta}$ in these conditions (Figure S3A). These experiments showed that there was a 2.2-fold increase in levels of phosphorylated ERK1/2 in the *Fgfr1*$^{\Delta/\Delta}$ cells. In addition, there was a 3.8-fold increase in total AKT protein and a 1.3-fold increase in the fraction of phosphorylated AKT (Figures 3B and 3C). The change in MAPK pathway activity was confirmed in vivo by immunostaining for pERK1/2, which showed higher levels in *Fgfr1*$^{\Delta/\Delta}$ BCs than controls at 1.5 weeks after tmx administration (Figure 3E). Thus, the loss of an RTK surprisingly resulted in increased levels of signaling through the RAS-ERK and PI3K intracellular pathways, which are typically associated with active RTK signaling. We therefore analyzed negative regulators of these signaling cascades. SPRY2 is a well-known transcriptional target of FGFR signaling and has previously been shown to be post-translationally modified downstream of FGFR1 activity in vitro (Lao et al., 2007; Rubin et al., 2005). Two isoforms of SPRY2 can be separated on polyacrylamide gels. Levels of the faster-migrating (lower) isoform have been shown in vitro to increase following FGFR1 activation. In addition, this isoform has a greater ability to bind to GRB2 in vitro and has been proposed to be the active isoform for inhibition of RAS-ERK signaling (Lao et al., 2007). Interestingly, in the *Fgfr1*$^{\Delta/\Delta}$ cells there was a 2.2-fold decrease in levels of the faster-migrating (lower, active for ERK/AKT inhibition) SPRY2 isoform and a 1.3-fold increase in levels of the slower-migrating (upper, unable to inhibit ERK/AKT) isoform (Figures 3B and 3C). This suggests that a wild-type function of FGFR1 signaling in the tracheal BCs is to modulate SPRY2 activity post-translationally, thus altering the ability of SPRY2 to inhibit signaling downstream of other RTKs (Figure 3D).

There is a strong connection between EGFR signaling and airway BC proliferation (Brechbuhl et al., 2014). We tested the hypothesis that because the *Fgfr1* cKO BCs have more constitutive activity of the ERK/AKT pathways, they would be more resistant to EGFR inhibition than controls. We grew *Fgfr1* cKO and control BCs in the presence of 0.25 μM of the EGFR inhibitor erlotinib (Figure S4A). This resulted in a >5-fold decrease in proliferation rates in the control cells, versus a 2-fold decrease in the *Fgfr1* cKO cells (Figures S4B–S4D). Moreover, levels of EGFR protein were unchanged in the *Fgfr1* cKO cells (Figure S4E). The *Fgfr1* cKO BCs are thus more resistant to EGFR inhibition, supporting the hypothesis that the downstream signaling pathways (ERK/PI3K) are more constitutively active in these cells.

Stimulating BCs with FGF2 Results in Increased Levels of the Faster-Migrating SPRY2 Isoform

We tested whether activating FGF signaling is sufficient to modulate levels of the SPRY2 isoforms in wild-type cells (Figure 3F). A 10-min or 24-hr FGF2 stimulation resulted in a reproducible small decrease in pERK1/2 compared with control cells, particularly at the 10-min harvest time (Figures 3G, 3H, and S5A–S5C). Following FGF2 stimulation we observed no change in total SPRY2 levels but a 1.6-fold increase in levels of the faster-migrating SPRY2 isoform (Figures 3G and 3H). These changes never reached statistical significance. However, the experiments were performed in primary cells that have multiple inputs into the ERK pathway in the presence of serum and the data were reproducible, supporting our hypothesis that manipulating FGFR1 signaling results in post-translational changes in SPRY2.

Fgfr1$^{\Delta/\Delta}$ BCs Cannot Produce Ciliated Cells

It has previously been reported that FGFR1 signaling in zebrafish and *Xenopus* embryos can regulate cilia assembly and length (Hong and Dawid, 2009; Neugebauer et al., 2009). We therefore examined ciliogenesis in the *Fgfr1* cKO tracheae. At 24 weeks after tmx administration, *Fgfr1* cKO tracheae had an excess of GFP$^+$ secretory cells and fewer GFP$^+$ ciliated cells than the controls (Figures 4A and 4B), suggesting that ciliated cell production was impaired in the mutant. Indeed, the only GFP$^+$ ciliated cells that could be detected in the *Fgfr1* cKO trachea still retained FGFR1 protein (Figures 4A and 4C), indicating that they were derived from *Fgfr1*$^{\Delta/fx}$ cells, which had undergone partial recombination to activate the *Rosa* reporter but had not deleted the *Fgfr1*^{fx} allele. Based on scoring the percentage of luminal GFP$^+$ cells that retained detectable FGFR1 protein in these samples, we were able to estimate the rate of co-recombination of the two floxed alleles as 84% ± 2% (Figure 4C), in good agreement with our qRT-PCR-based estimate (Figure 1C).

To assess the stage at which ciliated cell differentiation was blocked in the *Fgfr1* cKO cells, we stained tracheal sections for the transcription factor FOXJ1, which is expressed during ciliated cell differentiation and has a role in basal body anchoring (Blatt et al., 1999; Huang et al., 2003). In the *Fgfr1* cKO tracheae the number of GFP$^+$, FOXJ1$^+$ cells was significantly decreased, confirming an early block in ciliated cell differentiation (Figures 4B and 4D). These data suggest that in the *Fgfr1* cKO tracheae, luminal cell fate choice is disrupted and secretory cells are produced at the expense of ciliated cells. This phenotype is somewhat different to the published role for FGFR1 in controlling cilia length in multi-ciliated cells in the

(F) Schematic for exposure of wild-type BCs to FGF2.

(G) Representative western blots from day-6 FGF2-stimulated and control BCs showing SPRY2, pERK1/2, total ERK1/2, pAKT, total AKT, and histone H3.

(H) Quantification of protein levels in (G).

Error bars denote SEM. See also Figures S3–S5.

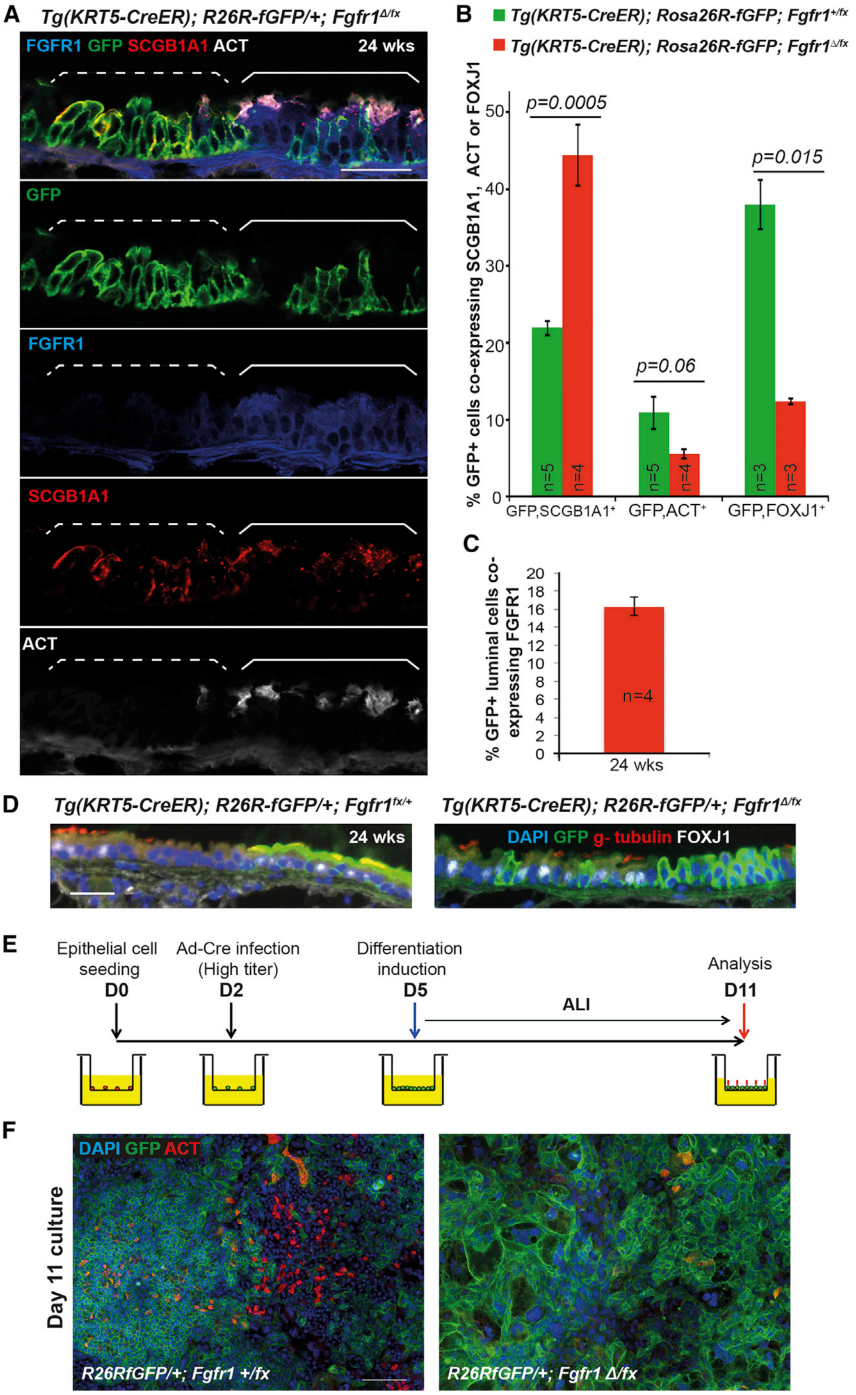

Figure 4. Loss of FGFR1 Signaling Results in a Block in Ciliated Cell Differentiation

(A) Confocal image of *Tg(KRT5-CreER)*; *Rosa26R*$^{fGFP/+}$; *Fgfr1*$^{\Delta/fx}$ tracheal section. Green, GFP (reporter); blue, FGFR1; red, SCGB1A1 (secretory cells); white, acetylated tubulin (ACT, cilia). In the patch of GFP^{+} cells marked by the dashed bracket, FGFR1 is absent and there are no ciliated cells. In the GFP^{+} cells within the solid bracket, FGFR1 has not been deleted, there are fewer GFP^{+} cells, and ciliated cell differentiation has occurred.

(B) Percentage of GFP^{+} cells that co-express SCGB1A1, ACT, or FOXJ1 in control *Tg(KRT5-CreER)*; *Rosa26R*$^{fGFP/+}$; *Fgfr1*$^{+/fx}$ and cKO *Tg(KRT5-CreER)*; *Rosa26R*$^{fGFP/+}$; *Fgfr1*$^{\Delta/fx}$ animals 24 weeks after tmx administration.

(C) Percentage of GFP^{+} luminal cells in cKO *Tg(KRT5-CreER)*; *Rosa26R*$^{fGFP/+}$; *Fgfr1*$^{\Delta/fx}$ trachea that retain FGFR1 protein 24 weeks after tmx administration.

(D) Sections from control and *Fgfr1* cKO tracheae at 24 weeks after tmx administration. Green, GFP (reporter); red, γ-tubulin (basal bodies); white, FOXJ1 (ciliated cells).

(E) Schematic of in vitro experiment.

(F) Day-11 ALI cultures grown from control and *Fgfr1* cKO animals. Green, GFP (reporter); red, ACT (cilia); blue, DAPI (nuclei).

Error bars denote SEM. Scale bars represent 100 μm in (A) and (F), and 50 μm in (D).

zebrafish and *Xenopus* systems (Hong and Dawid, 2009; Neugebauer et al., 2009). This may be a true cross-species difference or may reflect the limitations of the different experimental systems used.

Homeostatic production of new ciliated cells occurs at an extremely slow rate in vivo (Rawlins and Hogan, 2008). To confirm the ciliated cell differentiation phenotype in the *Fgfr1*$^{\Delta/\Delta}$ cells, we grew air-liquid interface (ALI) epithelial cultures from *Rosa26R-fGFP*; *Fgfr1*$^{\Delta/fx}$ and control *Rosa26R-fGFP*; *Fgfr1*$^{+/fx}$ tracheae and deleted the floxed alleles using Ad-Cre (Figure 4E). All control cultures had robust differentiation of ciliated cells, based on acetylated tubulin (ACT) localization, but ciliated cells were not observed in the *Fgfr1* cKO cultures (Figure 4F).

In summary, the *Fgfr1* cKO BCs have an increased proliferation rate from 1.5 weeks after injection. They are also unable to produce mature ciliated cells, likely due to a fate-choice defect. We focused on BC proliferation as the primary phenotype and, based on our observation that levels of the fast-migrating SPRY2 isoform were decreased in the *Fgfr1* cKO cells (Figures 3B and 3C), we hypothesized that a major role of FGFR1 signaling in the tracheal BCs is to modulate SPRY2 activity and, moreover, that the function of SPRY2 in these cells is to inhibit signaling activity downstream of RTKs other than FGFR1, for example EGFR, which is necessary for tracheal BC proliferation (Brechbuhl et al., 2014; Lu et al., 2013). To test this hypothesis, we deleted *Spry2* in BCs of the adult mouse trachea.

Loss of *Spry2* Results in Increased Levels of Tracheal Basal Cell Proliferation

Spry2 is widely expressed in basal and luminal cells of the homeostatic mouse trachea (Figures 1A and S1). We generated

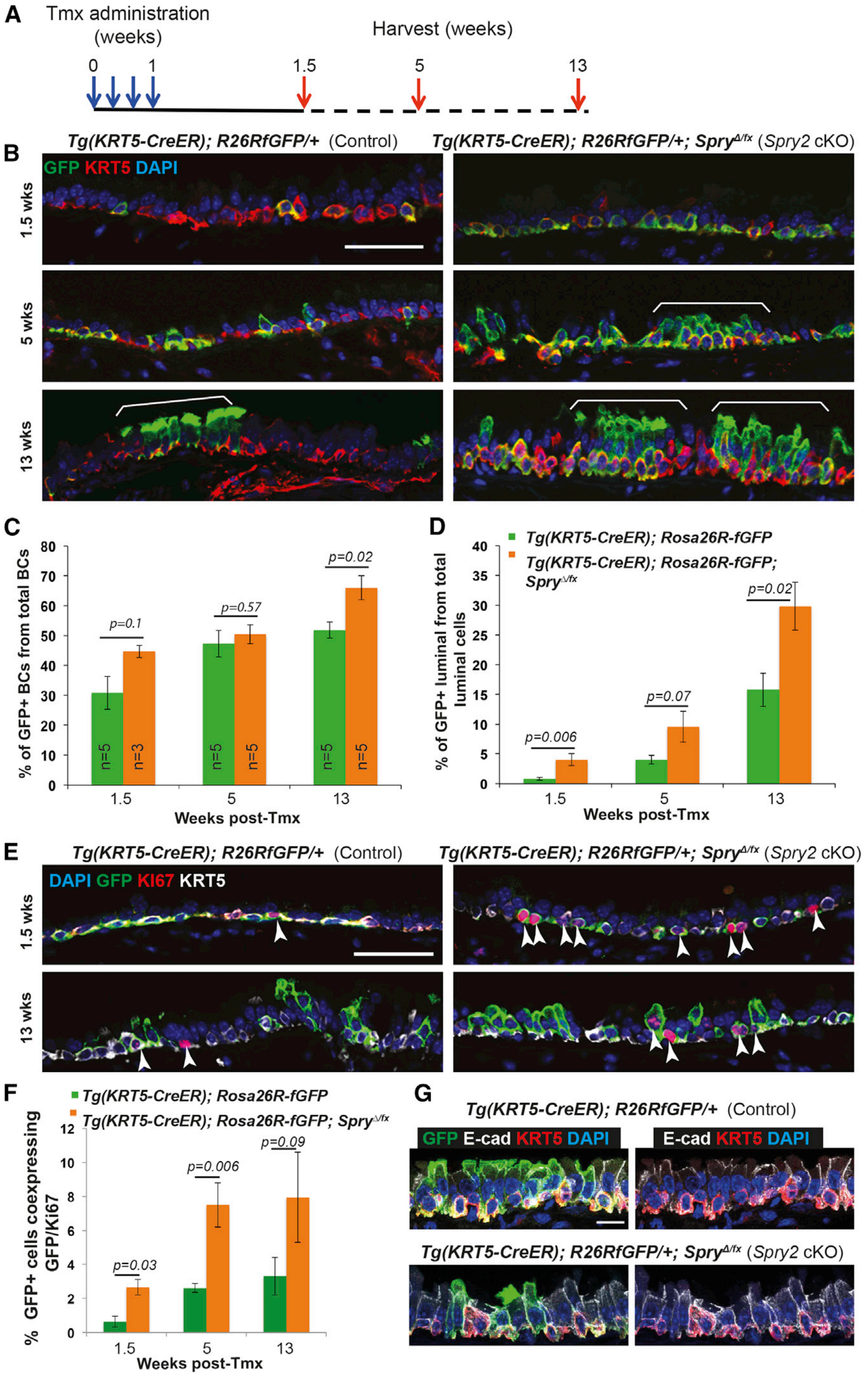

Figure 5. Deletion of *Spry2* in BCs Results in Increased Rates of Proliferation

(A) Schematic of *Spry2* conditional knockout experiment.

(B) Representative confocal sections from control *Tg(KRT5-CreER)*; *Rosa26R$^{fGFP/+}$* and cKO *Tg(KRT5-CreER)*; *Rosa26R$^{fGFP/+}$*; *Spry2$^{\Delta/fx}$* tracheae. Green, GFP (reporter); red, KRT5 (BCs); blue, DAPI (nuclei). Brackets indicate patches of labeled cells, more prominent in the *Spry2* cKO.

(C) Percentage of the total T1α^+ BCs that are also GFP$^+$.

(D) Percentage of the total T1α^- luminal cells that are also GFP$^+$.

(E) Representative confocal sections from control and cKO tracheae at 1.5 and 13 weeks after tmx administration. Green, GFP (reporter); red, KI67 (proliferating cells); white, KRT5 (BCs); blue, DAPI (nuclei). Arrowheads indicate KI67$^+$ cells.

(F) Percentage of GFP$^+$ cells that co-express KI67.

(G) Confocal z projections from sections of control and *Spry2 cKO* trachea. Green, GFP (reporter); red, KRT5 (BCs); white, E-cadherin (lateral cell membranes); blue, DAPI (nuclei).

Error bars denote SEM. Scale bars represent 50 μm in (B) and (E), and 10 μm in (G). See also Figure S6.

Tg(*KRT5-CreER*); *Rosa26R-fGFP*; *Spry2$^{\Delta/fx}$* (*Spry2* cKO) and control *Tg*(*KRT5-CreER*); *Rosa26R-fGFP* adult mice and administered tmx to activate GFP and delete *Spry2* in a subset of BCs (Figure 5A). During normal turnover there was an increase in the proportion of GFP$^+$, *Spry2$^{\Delta/\Delta}$* BCs compared with GFP$^+$, *Spry2$^{+/+}$* controls (Figures 5B and 5C). At the same time the proportion of GFP$^+$, *Spry2$^{\Delta/\Delta}$* luminal cells also increased (Figures 5B and 5D). These changes most likely resulted from the ~4-fold increase in rates of BC proliferation observed in the *Spry2* cKOs compared with controls (Figures 5E and 5F).

The proliferation phenotypes were thus very similar between the *Fgfr1* and *Spry2* cKO tracheae, supporting a strong connection between the two proteins. However, unlike the *Fgfr1* cKO, no effects on ciliated cell differentiation were observed in the *Spry2* cKO tracheae (Figures S6A and S6B), suggesting that the function of FGFR1 in luminal fate choice is SPRY2 independent. In addition, in contrast to *Fgfr1*, no phenotypes were observed in *Spry2* conditional heterozygous tracheae (data not shown).

The *Spry2* cKO cells were packed more tightly than control cells in the epithelium (at 13 weeks after tmx administration there were 16 ± 1.3 cells per 40 μm in the control tracheal epithelia versus 23.7 ± 1.6 cells per 40 μm in the *Spry2* cKOs; p = 0.01; Figure 5B), consistent with the significantly increased proliferation rate. However, there was no evidence of epithelial stratification, and levels of E-cadherin protein (Figure 5G) and apical-basal polarity (Figure S6C) appeared normal. These data suggest that the observed change in cell density may result from an altered threshold for contact inhibition in the *Spry2* cKO cells.

We tested whether we could recapitulate the *Spry2* cKO phenotypes using Ad-Cre in primary tracheal cell culture (Figure 6A). Deletion with a high titer of Ad-Cre resulted in loss of all detectable SPRY2 protein (Figure 6E) and failure to amplify the *Spry2^{fx}* allele from the genomic DNA (Figure S3B), suggesting that we

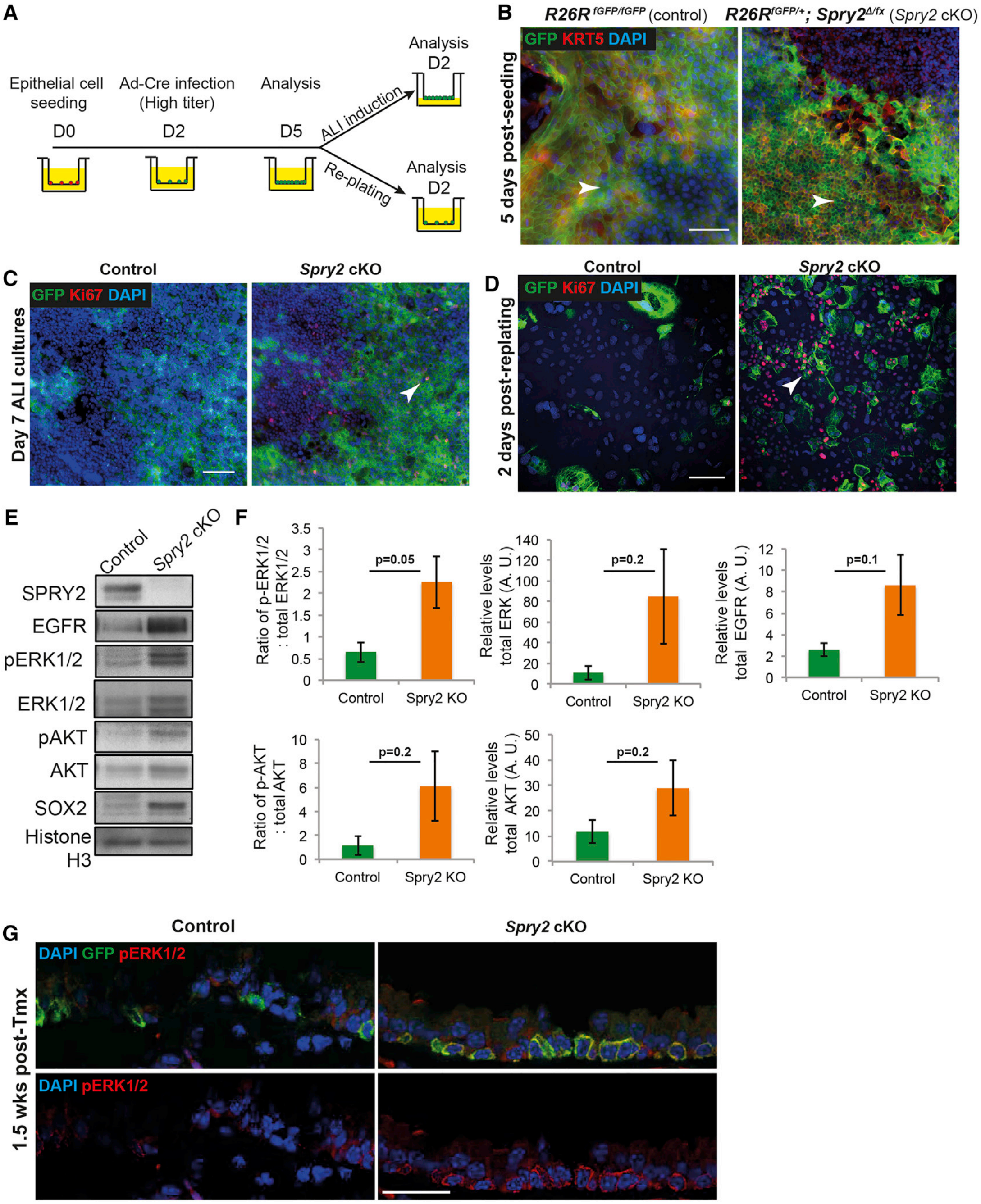

Figure 6. Rapidly Proliferating *Spry2* Conditional Knockout BCs Have Elevated Levels of RAS-ERK and PI3K Signaling

(A) Schematic of in vitro experiment.

(B) *Spry2* cKO (*Rosa26R*$^{fGFP/+}$; *Spry2*$^{\Delta/fx}$) cells proliferate more rapidly than controls (*Rosa26R*$^{fGFP/fGFP}$) in vitro and are more densely packed at culture day 5. Note that not all cKO cells are GFP^{+}, even though SPRY2 protein is undetectable. Green, GFP (reporter); red, KRT5 (BCs); blue, DAPI (nuclei). Arrowheads indicate similar regions of the cultures.

(C) *Spry2* cKO cells still undergo some contact inhibition in vitro at day 7 after ALI induction. Their proliferation rate slows following induction of ALI and there are no regions of multi-layering, although KI67^{+} cells are still visible (arrowhead). Green, GFP (reporter); red, KI67 (proliferating cells); blue, DAPI (nuclei).

(D) *Spry2* cKO primary cells continue to proliferate faster in vitro following passage at day 2 after replating. Green, GFP (reporter); red, KI67 (proliferating cells, e.g. arrowhead); blue, DAPI (nuclei).

(E) Representative western blots from control and *Spry2* cKO day-5 BCs showing SPRY2, pAKT, total AKT, pERK1/2, total ERK1/2, SOX2, and histone H3.

(F) Quantification of protein levels. Error bars denote SEM.

(G) Confocal images of control *Tg*(*KRT5-CreER*); *Rosa26R*$^{fGFP/+}$ and cKO *Tg*(*KRT5-CreER*); *Rosa26R*$^{fGFP/+}$; *Spry*$^{\Delta/fx}$ tracheal sections at 1.5 weeks after tmx administration showing an increase in pERK1/2 levels in the *Spry2* cKO BCs. Green, GFP (reporter); red, pERK1/2 (active ERK1/2); blue, DAPI (nuclei).

Scale bars, 25 μm. See also Figure S3.

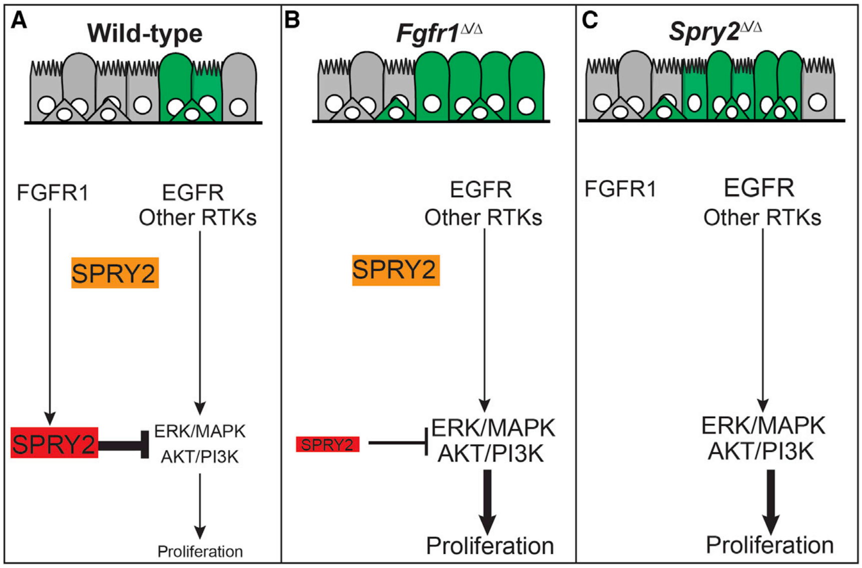

Figure 7. Model of the Roles of FGFR1 and SPRY2 in Airway Basal Cells

(A–C) Model for FGFR1-SPRY2 function in airway BCs. (A) In wild-type BCs, FGFR1 signaling post-translationally modifies SPRY2 resulting in an isoform of SPRY2 (red), which is able to inhibit ERK/MAPK and AKT/PI3K signaling downstream of other RTKs, such as EGFR. This FGFR1-SPRY2 signaling axis limits BC proliferation, resulting in low levels of steady-state production of new cells. (B) Following loss of FGFR1 there is a much smaller pool of ERK/AKT-inhibiting SPRY2 (red) and, hence, more active ERK/MAPK and AKT/PI3K signaling and greater levels of BC proliferation and production of new basal and luminal cells. In addition, the *Fgfr1*$^{Δ/Δ}$ cells cannot differentiate as mature multi-ciliated cells due to a role for FGFR1 signaling in luminal fate choice. (C) Similarly, following loss of all SPRY2 there is more active ERK/MAPK and AKT/PI3K signaling and greater levels of BC proliferation and, hence, production of new basal and luminal cells. Moreover, a second isoform of SPRY2 (orange), which may regulate the level of EGFR protein, is also lost. However, FGFR1 is present and ciliated cell differentiation is normal.

achieved complete recombination of the *Spry2*^{fx} allele. In spite of this there were large patches of GFP^{-} cells in our cultures that had not recombined the *Rosa* reporter (Figure 6B). This indicates that the *Spry2*^{fx} allele recombines more efficiently than the *Rosa26R-fGFP* reporter and supports the conclusion that most of the GFP^{+} cells observed in vivo were co-recombinants with *Spry2*^{fx}. Therefore, it is likely that scoring GFP^{+} cells in the *Spry2* cKO tracheae results in a good approximation to the *Spry2*$^{Δ/Δ}$ phenotype (compare with the *Fgfr1* cKO tracheae, where scoring GFP^{+} cells results in an underestimate of the *Fgfr1*$^{Δ/Δ}$ phenotype).

The *Spry2*$^{Δ/Δ}$ cultures had a greater rate of proliferation than the controls and also reached a higher cell density (Figures 6B and 6C). Proliferation of the *Spry2*$^{Δ/Δ}$ cells slowed after the cells reached confluence, but did continue (Figure 6C). Moreover, confluent cultures could easily be passaged and continued to show a faster proliferation rate (Figure 6D). Importantly, although the *Spry2*$^{Δ/Δ}$ cells reached a greater density than controls, we never observed phenotypes consistent with a complete loss of contact inhibition, such as multi-layering or cell detachment. This is again consistent with an altered threshold for, but not complete loss of, contact inhibition.

Similar to the *Fgfr1*$^{Δ/Δ}$ cells, the *Spry2*$^{Δ/Δ}$ cells had a 3.5-fold increase in pERK1/2 and a 5.2-fold increase in pAKT levels compared with the controls (Figures 6E and 6F). These changes were highly reproducible, although not always statistically significant. The increase in MAPK pathway activity was confirmed in vivo by immunostaining for pERK1/2, which showed higher levels in *Spry2*$^{Δ/Δ}$ BCs than controls at 1.5 weeks after tmx administration (Figure 6G). We also observed that the *Spry2*$^{Δ/Δ}$ cells had a 3.3-fold increase in levels of EGFR protein (Figures 6E and 6F). This is consistent with the published function of the slower-migrating SPRY2 isoform (which is absent in the *Spry2*$^{Δ/Δ}$ cells, but is unaffected in the *Fgfr1*$^{Δ/Δ}$ cells) in EGFR trafficking/degradation (Gao et al., 2012; Kim et al., 2007; Walsh and Lazzara, 2013).

DISCUSSION

The tracheal epithelium is normally quiescent, with a minimal amount of cell turnover. By combining in vivo genetic analysis and primary cell culture, we have shown that BC proliferation is actively repressed under steady-state conditions by an FGFR1-SPRY2 signaling axis that functions to inhibit signaling through the ERK and AKT intracellular pathways. This result argues that airway quiescence does not result simply from the absence of a proliferative signal or from feedback inhibitory loops. Rather, we propose a model in which quiescence is maintained by a balance between signaling mechanisms that either promote proliferation or repress it. This is an emerging theme in the stem cell biology of quiescent organs. For example, a recent publication has found a similar tonic Hedgehog signal that inhibits steady-state proliferation in the smaller mouse airways (Peng et al., 2015), but it is not known whether this Hedgehog-based mechanism applies in the BC-containing trachea.

SPRY2 was previously characterized in vitro as a protein with two isoforms, which can either inhibit ERK/AKT signaling (faster-migrating SPRY2 isoform) or modulate the subcellular trafficking/degradation of EGFR protein (slower-migrating isoform) (Guy et al., 2009). However, the relative roles of these SPRY2 isoforms in vivo and how they function downstream of RTK receptors have not previously been explored. Several lines of evidence presented here support a model in which the faster-migrating SPRY2 functions downstream of FGFR1 to control cell proliferation and tracheal epithelial homeostasis (Figure 7A). First, following the loss of FGFR1 protein in airway BCs we observe reduced levels of the faster-migrating (ERK/AKT-inhibiting) SPRY2 isoform, as well as increased levels of dpERK and pAKT, increased rates of proliferation, and increased resistance to EGFR inhibition. By contrast, the slower-migrating SPRY2 isoform is unaffected (Figure 7B). Second, exposing wild-type BCs to FGF2 ligand results in increased levels of

faster-migrating SPRY2, a decrease in dpERK, and inhibition of BC proliferation. Third, loss of all SPRY2 results in increased ERK/AKT signaling and BC proliferation (Figure 7C). Taken together, our data suggest that active FGFR1 signaling in the steady-state trachea maintains a pool of SPRY2 protein, which functions to antagonize ERK/AKT signaling and limit cell proliferation downstream of other RTKs, most likely EGFR (Figure 7A). A relationship between FGFR1 and post-translational modification of SPRY2 protein has previously been documented in cell lines in vitro (Lao et al., 2006, 2007). We now demonstrate its importance in vivo, clearly illustrating that the context in which a cell interprets EGFR/RTK signaling can be highly dependent on the activity of other RTKs present. This has clear implications for cancer development and the evolution of drug-resistant tumors.

Another important finding of our work is that FGFR1 and SPRY2 can also function independently in airway epithelial homeostasis. FGFR1 is required for multi-ciliated cell differentiation (Figure 4). Moreover, our data suggest that secretory cells are produced at the expense of ciliated cells by the *Fgfr1* cKO BCs. A role for FGFR1 in ciliated cell differentiation is also consistent with previous reports from other model organisms (Hong and Dawid, 2009; Neugebauer et al., 2009), but the details of the cellular requirements remain to be worked out. SPRY2 regulates EGFR protein levels in BCs (Figure 6). Similar observations have previously been made in vitro or in cancer models, but we demonstrate this SPRY2-EGFR relationship for the first time in a homeostatic tissue (Gao et al., 2012; Kim et al., 2007; Walsh and Lazzara, 2013). SPRY2 thus emerges as a highly flexible switch that can be modulated by FGFR1 signaling to control different aspects of RTK signaling. In excess of ten phosphorylation sites and multiple binding partners have been documented for SPRY2 in vitro (DaSilva et al., 2006; Edwin et al., 2009; Lao et al., 2007). These data suggest that SPRY2 is extensively and flexibly modified in many different cellular settings in vivo and that our observations may be the tip of the iceberg for the control of cellular homeostasis by SPRY2.

A key question raised by our model concerns what activates FGFR1 in vivo. We observed that FGF2 is transcribed in the tracheal mesenchyme in vivo at steady state, and that recombinant FGF2 inhibits tracheal BC proliferation and modulates SPRY2 in vitro. These data are consistent with a role for FGF2 in activating FGFR1 signaling in vivo, although we note that other FGF ligands are expressed in the steady-state airways. Interestingly, in a recent screen of secreted proteins for their effect on the ability of human airway BCs to form clonal organoids in culture, addition of FGF2 resulted in smaller tracheospheres with fewer BCs (Danahay et al., 2015). This is highly consistent with our in vitro results and suggests that a role for FGF2 signaling in inhibiting airway BC proliferation is conserved between mouse and humans.

Amplification of the *FGFR1* locus, or increased levels of FGFR1 protein, have been observed in 10%–20% of squamous lung cancer cell lines, where FGFR1 has been implicated as a driver mutation and putative therapeutic target (Dutt et al., 2011; Weiss et al., 2010; Wynes et al., 2014). How do we reconcile this increase in FGFR1 expression in airway cancer with our finding that an FGFR1-SPRY2 signaling module is required to limit mouse airway BC proliferation at steady state? This will clearly require future investigation. However, the squamous cell cancer lines used in these studies were isolated from the end stage of a cancer development process and have undergone many genetic and epigenetic changes. Given the highly context-dependent and cell-type-specific nature of FGFR1 signaling, and its interaction with SPRY2 and other RTKs as we have observed, its function may change drastically during neoplasia development. Nevertheless, we note that if a normal function of FGFR1 signaling is to inhibit proliferation of wild-type airway BCs, then it is possible that administration of FGFR1-specific inhibitors as a cancer therapy may lead to clonal expansion of other pre-malignant cells.

In conclusion, airway epithelial quiescence requires active inhibition of proliferation by FGFR1 signaling, which results in the post-translational modification of SPRY2 and inhibition of signaling downstream of other RTKs, partly EGFR. We have thus demonstrated a specific in vivo function for an FGFR1-SPRY2 signaling axis that was previously documented in vitro. This illustrates the complexity of RTK signaling networks and the importance of further investigation in both homeostatic tissues and developing malignancies.

EXPERIMENTAL PROCEDURES

See Supplemental Experimental Procedures for a full description of all materials and methods used.

Mice

Experiments were performed under Home Office licenses PPL80/2326 and 70/812. *Fgfr1*^{fx} (Xu et al., 2002), *Tg*(*KRT5-CreER*) (Rock et al., 2009), *Rosa26R-fGFP* (Rawlins et al., 2009), *Spry2*^{fx} (Shim et al., 2005) were described previously. *Fgfr1*$^{\Delta/+}$ and *Spry2*$^{\Delta/+}$ were generated by crossing floxed alleles to *Zp3-Cre* (de Vries et al., 2000). All strains were maintained on a C57Bl/6J background. Males and females >8 weeks old were used. Wild-type mice were C57Bl/6J.

Tamoxifen

Animals were injected intraperitoneally with tmx, 0.2 mg/g body weight, four times every other day.

Tracheal Epithelial Cell Culture

Tracheae were cut into small pieces and incubated in 50% Dispase II (Gibco, 16 U/ml). Epithelial sheets were isolated and dissociated to single cells. Unless otherwise stated, 5 × 10^4 cells in 0.5 ml of MTEC/+ medium (You et al., 2002) were plated on collagen-coated 12-well tissue culture inserts (BD Falcon, 353180). Differentiation was induced in confluent cultures by removal of insert medium and addition of MTEC serum-free medium to the outer chamber. Ad-Cre (University of Iowa, Gene Transfer Vector Core) MOI 7 was used for scattered recombination and MOI 2,500 for complete recombination; 1 × 10^6 vector plaque-forming units, 100 ng/ml recombinant mouse FGF2, and 0.25 μM erlotinib (Sigma).

Immunostaining

Cryosections (6 μm) or whole-mount primary cultures were stained with ACT (mouse, 1:3,000, Sigma, T7451), E-cadherin (rat, 1:3,000, Invitrogen, 13-1900), pERK1/2 (rabbit, 1:200, Cell Signaling Technology, 4695), FGFR1 (rabbit, 1:200, Novus Biologicals, NBP1-20067), FOXJ1 (mouse, 1:200, eBioscience, clone 2A5, 14-9965-80), GFP (chick, 1:1,000, Abcam, Ab13970), KRT5 (rabbit, 1:500, Covance, PRB-160P), KI67 (mouse, 1:200, Becton Dickinson, 550609), SCGB1A1 (goat, 1:400, Santa Cruz Biotechnology, sc9772), SPRY2 (rabbit, 1:200, Abcam, Ab50317), and T1α (1:1,000, DSHB, 8.1.1). Antigen retrieval was in 10 mM boiling sodium citrate for KI67 (pH 8), pERK1/2 (pH 6.5), and FOXJ1 (pH 6). Conjugated secondary antibodies were Phalloidin-568 and Alexa Fluor (Life Technologies, 1:2,000).

mRNA In Situ Hybridization

Cryosections (10 μm) were hybridized with DIG-labeled anti-sense mRNA probes to *Fgfr1* (EST: AW495528) or *Spry2* (Minowada et al., 1999) (DIG RNA labeling kit, Roche, 11175025910), and visualized using alkaline phosphatase-coupled anti-DIG (1:2,000, Roche, 12930023) and BCIP/NBT (Roche, 1383213).

Microscopy and Image Scoring

Slides were imaged on a Zeiss AxioImager compound microscope or a Leica Sp8/Sp5 confocal microscope as stated. Cells were scored manually in Fiji (ImageJ), or using the Gurdon Institute Imaging Facility's plugin ObjectScan. For cryosections, every epithelial cell along the entire proximal to distal length of a longitudinal section from the center of the trachea was scored. BCs were $T1\alpha^+$ or $KRT5^+$ with the apical surface not reaching the lumen. For cultured cells, at least three random fields of view per insert were scored.

RT-PCR

Samples were enriched for epithelium or mesenchyme using Dispase digestion and manual peeling. Total RNA was extracted using TRIzol reagent.

qRT-PCR

Primary tracheal epithelial cells were isolated and sorted using a fluorescence-activated cell sorting MoFlo flow cytometer. Wild-type cells were gated as basal EpCAM, GSIβ4 lectin; secretory EpCAM, SSEA1; and ciliated EpCAM, CD24. GFP^+ BCs from control and *Fgfr1* cKO tracheae were sorted as GFP, GSIβ4 lectin. Total RNA was extracted using a Qiagen RNEasy Mini Kit, and Taqman gene expression assays (Life Technologies) were used.

Immunoblots

Cells were collected in Cell Extraction Buffer (Invitrogen, FNN0011) with protease inhibitor (Roche, 04693116001) and PMSF (Sigma, P7626). Proteins were separated on 10% or 12% SDS-PAGE gels before transfer to Millipore Immobilon-P PVDF Membrane (Merck Millipore, IPVH00010). Membranes were probed with pAKT(S473) (Cell Signaling, 3787, 1:3,000), pan-AKT (Cell Signaling, 4691, 1:1,000), dpERK1/2 (Cell Signaling, 4370, 1:300), ERK1/2 (Cell Signaling, 4695, 1:300), SPRY2 (Abcam, Ab50317, 1:300), SOX2 (Abcam, Ab97959, 1:3,000), Histone H3 (Abcam, Ab39655, 1:10,000), and EGFR (Millipore, 04-290, 1:300). Detection was performed with horseradish peroxidase-conjugated secondaries (Abcam, ab97051, ab97023, 1:10,000) and enhanced chemiluminescence (Thermo Scientific, PI-32109). Quantitation is based on analysis of protein from three biological replicates separated on the same polyacrylamide gel. Band intensity was analyzed in Fiji normalized to loading control.

Statistics

Statistics were calculated using Student's two-tailed t test with unequal variance.

SUPPLEMENTAL INFORMATION

Supplemental Information includes Supplemental Experimental Procedures and six figures and can be found with this article online at http://dx.doi.org/10.1016/j.devcel.2016.03.001.

AUTHOR CONTRIBUTIONS

G.B. designed and performed experiments, analyzed data, and edited the manuscript. J.J. performed experiments. M.A.B. provided mice and trachea samples and assisted with experimental design. E.R. conceived and led the project, performed experiments, analyzed data, and wrote and edited the manuscript.

ACKNOWLEDGMENTS

This study was supported by the Medical Research Council (G0900424 to E.R.) and a Wellcome Trust clinical PhD fellowship (J.J.). Core grants: Gurdon Institute: Wellcome Trust (092096), Cancer Research UK (C6946/A14492); Stem Cell Initiative: Wellcome Trust/MRC.

Received: July 21, 2015
Revised: January 6, 2016
Accepted: March 1, 2016
Published: April 4, 2016

REFERENCES

Abler, L.L., Mansour, S.L., and Sun, X. (2009). Conditional gene inactivation reveals roles for Fgf10 and Fgfr2 in establishing a normal pattern of epithelial branching in the mouse lung. Dev. Dyn. *238*, 1999–2013.

Arai, D., Hegab, A.E., Soejima, K., Kuroda, A., Ishioka, K., Yasuda, H., Naoki, K., Kagawa, S., Hamamoto, J., Yin, Y., et al. (2015). Characterization of the cell of origin and propagation potential of the fibroblast growth factor 9-induced mouse model of lung adenocarcinoma. J. Pathol. *235*, 593–605.

Blatt, E.N., Yan, X.H., Wuerffel, M.K., Hamilos, D.L., and Brody, S.L. (1999). Forkhead transcription factor HFH-4 expression is temporally related to ciliogenesis. Am. J. Respir. Cell Mol. Biol. *21*, 168–176.

Brechbuhl, H.M., Li, B., Smith, R.W., and Reynolds, S.D. (2014). Epidermal growth factor receptor activity is necessary for mouse basal cell proliferation. Am. J. Physiol. Lung Cell Mol. Physiol. *307*, L800–L810.

Chakkalakal, J.V., Jones, K.M., Basson, M.A., and Brack, A.S. (2012). The aged niche disrupts muscle stem cell quiescence. Nature *490*, 355–360.

Cole, B.B., Smith, R.W., Jenkins, K.M., Graham, B.B., Reynolds, P.R., and Reynolds, S.D. (2010). Tracheal basal cells: a facultative progenitor cell pool. Am. J. Pathol. *177*, 362–376.

Danahay, H., Pessotti, A.D., Coote, J., Montgomery, B.E., Xia, D., Wilson, A., Yang, H., Wang, Z., Bevan, L., Thomas, C., et al. (2015). Notch2 is required for inflammatory cytokine-driven goblet cell metaplasia in the lung. Cell Rep. *10*, 239–252.

DaSilva, J., Xu, L., Kim, H.J., Miller, W.T., and Bar-Sagi, D. (2006). Regulation of sprouty stability by Mnk1-dependent phosphorylation. Mol. Cell Biol. *26*, 1898–1907.

de Vries, W.N., Binns, L.T., Fancher, K.S., Dean, J., Moore, R., Kemler, R., and Knowles, B.B. (2000). Expression of Cre recombinase in mouse oocytes: a means to study maternal effect genes. Genesis *26*, 110–112.

Dutt, A., Ramos, A.H., Hammerman, P.S., Mermel, C., Cho, J., Sharifnia, T., Chande, A., Tanaka, K.E., Stransky, N., Greulich, H., et al. (2011). Inhibitor-sensitive FGFR1 amplification in human non-small cell lung cancer. PLoS One *6*, e20351.

Edwin, F., Anderson, K., Ying, C., and Patel, T.B. (2009). Intermolecular interactions of Sprouty proteins and their implications in development and disease. Mol. Pharmacol. *76*, 679–691.

Gao, M., Patel, R., Ahmad, I., Fleming, J., Edwards, J., McCracken, S., Sahadevan, K., Seywright, M., Norman, J., Sansom, O., et al. (2012). SPRY2 loss enhances ErbB trafficking and PI3K/AKT signalling to drive human and mouse prostate carcinogenesis. EMBO Mol. Med. *4*, 776–790.

Garmy-Susini, B., Delmas, E., Gourdy, P., Zhou, M., Bossard, C., Bugler, B., Bayard, F., Krust, A., Prats, A.C., Doetschman, T., et al. (2004). Role of fibroblast growth factor-2 isoforms in the effect of estradiol on endothelial cell migration and proliferation. Circ. Res. *94*, 1301–1309.

Giangreco, A., Lu, L., Vickers, C., Teixeira, V.H., Groot, K.R., Butler, C.R., Ilieva, E.V., George, P.J., Nicholson, A.G., Sage, E.K., et al. (2012). Beta-catenin determines upper airway progenitor cell fate and preinvasive squamous lung cancer progression by modulating epithelial-mesenchymal transition. J. Pathol. *226*, 575–587.

Guy, G.R., Jackson, R.A., Yusoff, P., and Chow, S.Y. (2009). Sprouty proteins: modified modulators, matchmakers or missing links? J. Endocrinol. *203*, 191–202.

Hackett, N.R., Shaykhiev, R., Walters, M.S., Wang, R., Zwick, R.K., Ferris, B., Witover, B., Salit, J., and Crystal, R.G. (2011). The human airway epithelial basal cell transcriptome. PLoS One *6*, e18378.

Hadjab, S., Franck, M.C., Wang, Y., Sterzenbach, U., Sharma, A., Ernfors, P., and Lallemend, F. (2013). A local source of FGF initiates development of the unmyelinated lineage of sensory neurons. J. Neurosci. *33*, 17656–17666.

Hogan, B.L., Barkauskas, C.E., Chapman, H.A., Epstein, J.A., Jain, R., Hsia, C.C., Niklason, L., Calle, E., Le, A., Randell, S.H., et al. (2014). Repair and regeneration of the respiratory system: complexity, plasticity, and mechanisms of lung stem cell function. Cell Stem Cell *15*, 123–138.

Hong, S.K., and Dawid, I.B. (2009). FGF-dependent left-right asymmetry patterning in zebrafish is mediated by Ier2 and Fibp1. Proc. Natl. Acad. Sci. USA *106*, 2230–2235.

Hong, K.U., Reynolds, S.D., Watkins, S., Fuchs, E., and Stripp, B.R. (2004). In vivo differentiation potential of tracheal basal cells: evidence for multipotent and unipotent subpopulations. Am. J. Physiol. Lung Cell Mol. Physiol. *286*, L643–L649.

Huang, T., You, Y., Spoor, M.S., Richer, E.J., Kudva, V.V., Paige, R.C., Seiler, M.P., Liebler, J.M., Zabner, J., Plopper, C.G., et al. (2003). Foxj1 is required for apical localization of ezrin in airway epithelial cells. J. Cell Sci. *116*, 4935–4945.

Kauffman, S.L. (1980). Cell proliferation in the mammalian lung. Int. Rev. Exp. Pathol. *22*, 131–191.

Kim, H.J., Taylor, L.J., and Bar-Sagi, D. (2007). Spatial regulation of EGFR signaling by Sprouty2. Curr. Biol. *17*, 455–461.

Kranenburg, A.R., Willems-Widyastuti, A., Mooi, W.J., Saxena, P.R., Sterk, P.J., de Boer, W.I., and Sharma, H.S. (2005). Chronic obstructive pulmonary disease is associated with enhanced bronchial expression of FGF-1, FGF-2, and FGFR-1. J. Pathol. *206*, 28–38.

Lao, D.H., Chandramouli, S., Yusoff, P., Fong, C.W., Saw, T.Y., Tai, L.P., Yu, C.Y., Leong, H.F., and Guy, G.R. (2006). A Src homology 3-binding sequence on the C terminus of Sprouty2 is necessary for inhibition of the Ras/ERK pathway downstream of fibroblast growth factor receptor stimulation. J. Biol. Chem. *281*, 29993–30000.

Lao, D.H., Yusoff, P., Chandramouli, S., Philp, R.J., Fong, C.W., Jackson, R.A., Saw, T.Y., Yu, C.Y., and Guy, G.R. (2007). Direct binding of PP2A to Sprouty2 and phosphorylation changes are a prerequisite for ERK inhibition downstream of fibroblast growth factor receptor stimulation. J. Biol. Chem. *282*, 9117–9126.

Lu, L., Teixeira, V.H., Yuan, Z., Graham, T.A., Endesfelder, D., Kolluri, K., Al-Juffali, N., Hamilton, N., Nicholson, A.G., Falzon, M., et al. (2013). LRIG1 regulates cadherin-dependent contact inhibition directing epithelial homeostasis and pre-invasive squamous cell carcinoma development. J. Pathol. *229*, 608–620.

Mahoney, J.E., Mori, M., Szymaniak, A.D., Varelas, X., and Cardoso, W.V. (2014). The hippo pathway effector Yap controls patterning and differentiation of airway epithelial progenitors. Dev. Cell *30*, 137–150.

Minowada, G., Jarvis, L.A., Chi, C.L., Neubuser, A., Sun, X., Hacohen, N., Krasnow, M.A., and Martin, G.R. (1999). Vertebrate Sprouty genes are induced by FGF signaling and can cause chondrodysplasia when overexpressed. Development *126*, 4465–4475.

Mori, M., Mahoney, J.E., Stupnikov, M.R., Paez-Cortez, J.R., Szymaniak, A.D., Varelas, X., Herrick, D.B., Schwob, J., Zhang, H., and Cardoso, W.V. (2015). Notch3-Jagged signaling controls the pool of undifferentiated airway progenitors. Development *142*, 258–267.

Neugebauer, J.M., Amack, J.D., Peterson, A.G., Bisgrove, B.W., and Yost, H.J. (2009). FGF signalling during embryo development regulates cilia length in diverse epithelia. Nature *458*, 651–654.

Ornitz, D.M., Xu, J., Colvin, J.S., McEwen, D.G., MacArthur, C.A., Coulier, F., Gao, G., and Goldfarb, M. (1996). Receptor specificity of the fibroblast growth factor family. J. Biol. Chem. *271*, 15292–15297.

Pardo-Saganta, A., Law, B.M., Tata, P.R., Villoria, J., Saez, B., Mou, H., Zhao, R., and Rajagopal, J. (2015). Injury induces direct lineage segregation of functionally distinct airway basal stem/progenitor cell subpopulations. Cell Stem Cell *16*, 184–197.

Paul, M.K., Bisht, B., Darmawan, D.O., Chiou, R., Ha, V.L., Wallace, W.D., Chon, A.T., Hegab, A.E., Grogan, T., Elashoff, D.A., et al. (2014). Dynamic changes in intracellular ROS levels regulate airway basal stem cell homeostasis through Nrf2-dependent notch signaling. Cell Stem Cell *15*, 199–214.

Peng, T., Frank, D.B., Kadzik, R.S., Morley, M.P., Rathi, K.S., Wang, T., Zhou, S., Cheng, L., Lu, M.M., and Morrisey, E.E. (2015). Hedgehog actively maintains adult lung quiescence and regulates repair and regeneration. Nature *526*, 578–582.

Rawlins, E.L., and Hogan, B.L. (2008). Ciliated epithelial cell lifespan in the mouse trachea and lung. Am. J. Physiol. Lung Cell Mol. Physiol. *295*, L231–L234.

Rawlins, E.L., Ostrowski, L.E., Randell, S.H., and Hogan, B.L. (2007). Lung development and repair: contribution of the ciliated lineage. Proc. Natl. Acad. Sci. USA *104*, 410–417.

Rawlins, E.L., Okubo, T., Xue, Y., Brass, D.M., Auten, R.L., Hasegawa, H., Wang, F., and Hogan, B.L. (2009). The role of Scgb1a1+ Clara cells in the long-term maintenance and repair of lung airway, but not alveolar, epithelium. Cell Stem Cell *4*, 525–534.

Rock, J.R., Onaitis, M.W., Rawlins, E.L., Lu, Y., Clark, C.P., Xue, Y., Randell, S.H., and Hogan, B.L. (2009). Basal cells as stem cells of the mouse trachea and human airway epithelium. Proc. Natl. Acad. Sci. USA *106*, 12771–12775.

Rock, J.R., Randell, S.H., and Hogan, B.L. (2010). Airway basal stem cells: a perspective on their roles in epithelial homeostasis and remodeling. Dis. Model. Mech. *3*, 545–556.

Rock, J.R., Gao, X., Xue, Y., Randell, S.H., Kong, Y.Y., and Hogan, B.L. (2011). Notch-dependent differentiation of adult airway basal stem cells. Cell Stem Cell *8*, 639–648.

Rubin, C., Zwang, Y., Vaisman, N., Ron, D., and Yarden, Y. (2005). Phosphorylation of carboxyl-terminal tyrosines modulates the specificity of Sprouty-2 inhibition of different signaling pathways. J. Biol. Chem. *280*, 9735–9744.

Shea, K.L., Xiang, W., LaPorta, V.S., Licht, J.D., Keller, C., Basson, M.A., and Brack, A.S. (2010). Sprouty1 regulates reversible quiescence of a self-renewing adult muscle stem cell pool during regeneration. Cell Stem Cell *6*, 117–129.

Shim, K., Minowada, G., Coling, D.E., and Martin, G.R. (2005). Sprouty2, a mouse deafness gene, regulates cell fate decisions in the auditory sensory epithelium by antagonizing FGF signaling. Dev. Cell *8*, 553–564.

Teixeira, V.H., Nadarajan, P., Graham, T.A., Pipinikas, C.P., Brown, J.M., Falzon, M., Nye, E., Poulsom, R., Lawrence, D., Wright, N.A., et al. (2013). Stochastic homeostasis in human airway epithelium is achieved by neutral competition of basal cell progenitors. Elife *2*, e00966.

Vermeer, P.D., Einwalter, L.A., Moninger, T.O., Rokhlina, T., Kern, J.A., Zabner, J., and Welsh, M.J. (2003). Segregation of receptor and ligand regulates activation of epithelial growth factor receptor. Nature *422*, 322–326.

Volckaert, T., Dill, E., Campbell, A., Tiozzo, C., Majka, S., Bellusci, S., and De Langhe, S.P. (2011). Parabronchial smooth muscle constitutes an airway epithelial stem cell niche in the mouse lung after injury. J. Clin. Invest. *121*, 4409–4419.

Volckaert, T., Campbell, A., Dill, E., Li, C., Minoo, P., and De Langhe, S. (2013). Localized Fgf10 expression is not required for lung branching morphogenesis but prevents differentiation of epithelial progenitors. Development *140*, 3731–3742.

Walsh, A.M., and Lazzara, M.J. (2013). Regulation of EGFR trafficking and cell signaling by Sprouty2 and MIG6 in lung cancer cells. J. Cell Sci. *126*, 4339–4348.

Watson, J.K., Rulands, S., Wilkinson, A.C., Wuidart, A., Ousset, M., Van Keymeulen, A., Gottgens, B., Blanpain, C., Simons, B.D., and Rawlins, E.L. (2015). Clonal dynamics reveal two distinct populations of basal cells in slow-turnover airway epithelium. Cell Rep. *12*, 90–101.

Weiss, J., Sos, M.L., Seidel, D., Peifer, M., Zander, T., Heuckmann, J.M., Ullrich, R.T., Menon, R., Maier, S., Soltermann, A., et al. (2010). Frequent and focal FGFR1 amplification associates with therapeutically tractable FGFR1 dependency in squamous cell lung cancer. Sci. Transl. Med. *2*, 62ra93.

Wynes, M.W., Hinz, T.K., Gao, D., Martini, M., Marek, L.A., Ware, K.E., Edwards, M.G., Bohm, D., Perner, S., Helfrich, B.A., et al. (2014). FGFR1

mRNA and protein expression, not gene copy number, predict FGFR TKI sensitivity across all lung cancer histologies. Clin. Cancer Res. *20*, 3299–3309.

Xu, X., Qiao, W., Li, C., and Deng, C.X. (2002). Generation of Fgfr1 conditional knockout mice. Genesis *32*, 85–86.

Yin, Y., Wang, F., and Ornitz, D.M. (2011). Mesothelial- and epithelial-derived FGF9 have distinct functions in the regulation of lung development. Development *138*, 3169–3177.

You, Y., Richer, E.J., Huang, T., and Brody, S.L. (2002). Growth and differentiation of mouse tracheal epithelial cells: selection of a proliferative population. Am. J. Physiol. Lung Cell Mol. Physiol. *283*, L1315–L1321.

Zhao, R., Fallon, T.R., Saladi, S.V., Pardo-Saganta, A., Villoria, J., Mou, H., Vinarsky, V., Gonzalez-Celeiro, M., Nunna, N., Hariri, L.P., et al. (2014). Yap tunes airway epithelial size and Architecture by regulating the identity, maintenance, and self-Renewal of stem cells. Dev. Cell *30*, 151–165.

Developmental Cell, Volume *37*

Supplemental Information

An FGFR1-SPRY2 Signaling Axis Limits Basal Cell Proliferation in the Steady-State Airway Epithelium

Gayan I. Balasooriya, Jo-Anne Johnson, M. Albert Basson, and Emma L. Rawlins

Supplemental Information Inventory

Figure S1, related to Figure 1. *Fgfr1* and *Spry2* are expressed in tracheal basal cells.

Figure S2, related to Figures 1 and 2. *Fgfr1* heterozygous basal cells display a similar phenotype to conditional knock-out basal cells.

Figure S3, related to Figures 3 and 6. Recombination of $Fgfr1^{fx}$ and $Spry2^{fx}$ in vitro.

Figure S4, related to Figure 3. *Fgfr1* cKO basal cells are more resistant to EGFR signalling inhibition.

Figure S5, related to Figure 3. FGF2 stimulation of wild-type basal cells results in a small drop in pERK1/2 levels.

Figure S6, related to Figure 5. Ciliated cell differentiation and epithelial organisation are normal in the *Spry2* conditional knock-outs.

Supplemental Experimental Procedures. Full description of all materials and methods used.

Balasooriya et al. Figure S1

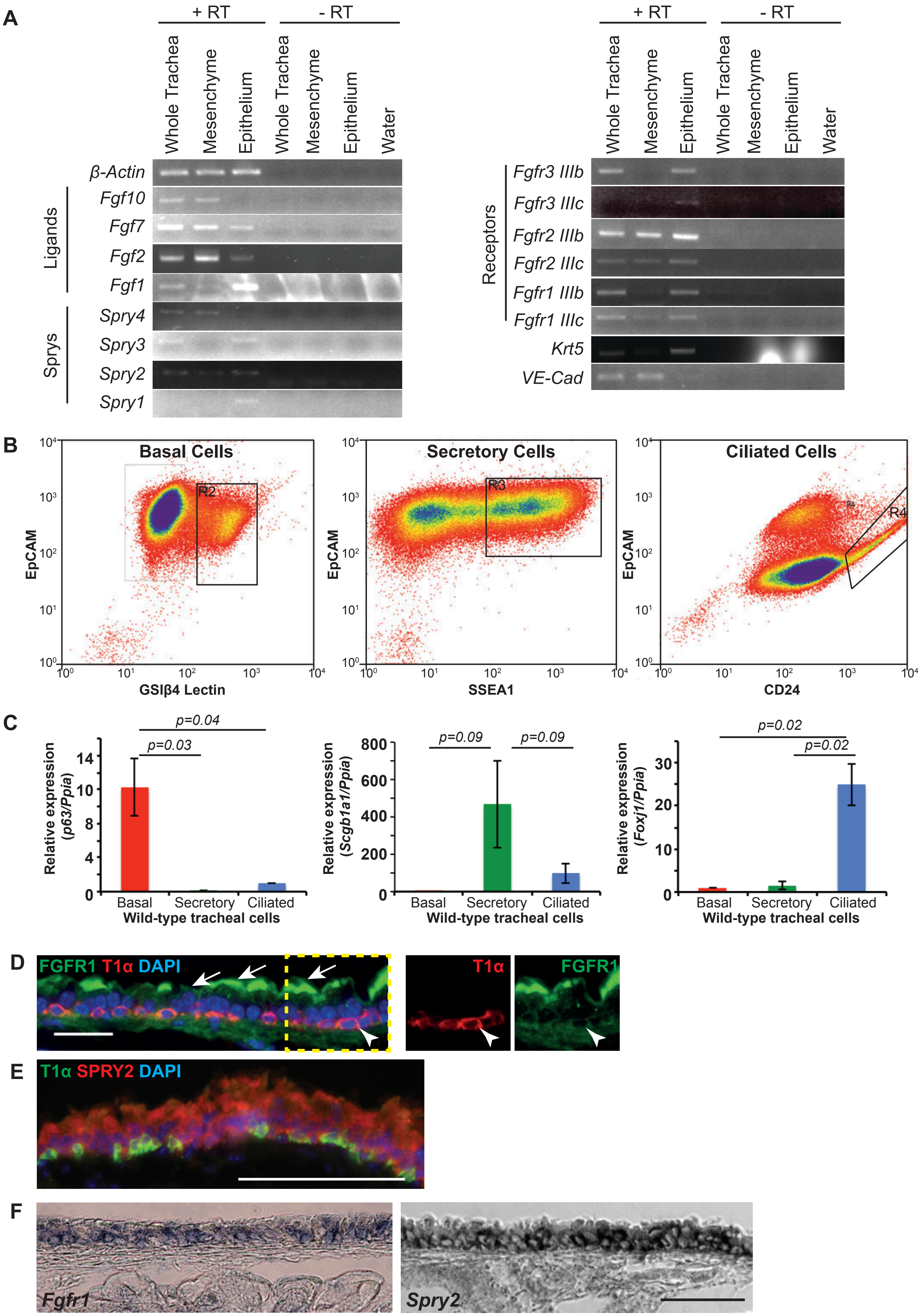

Balasooriya et al. Figure S2

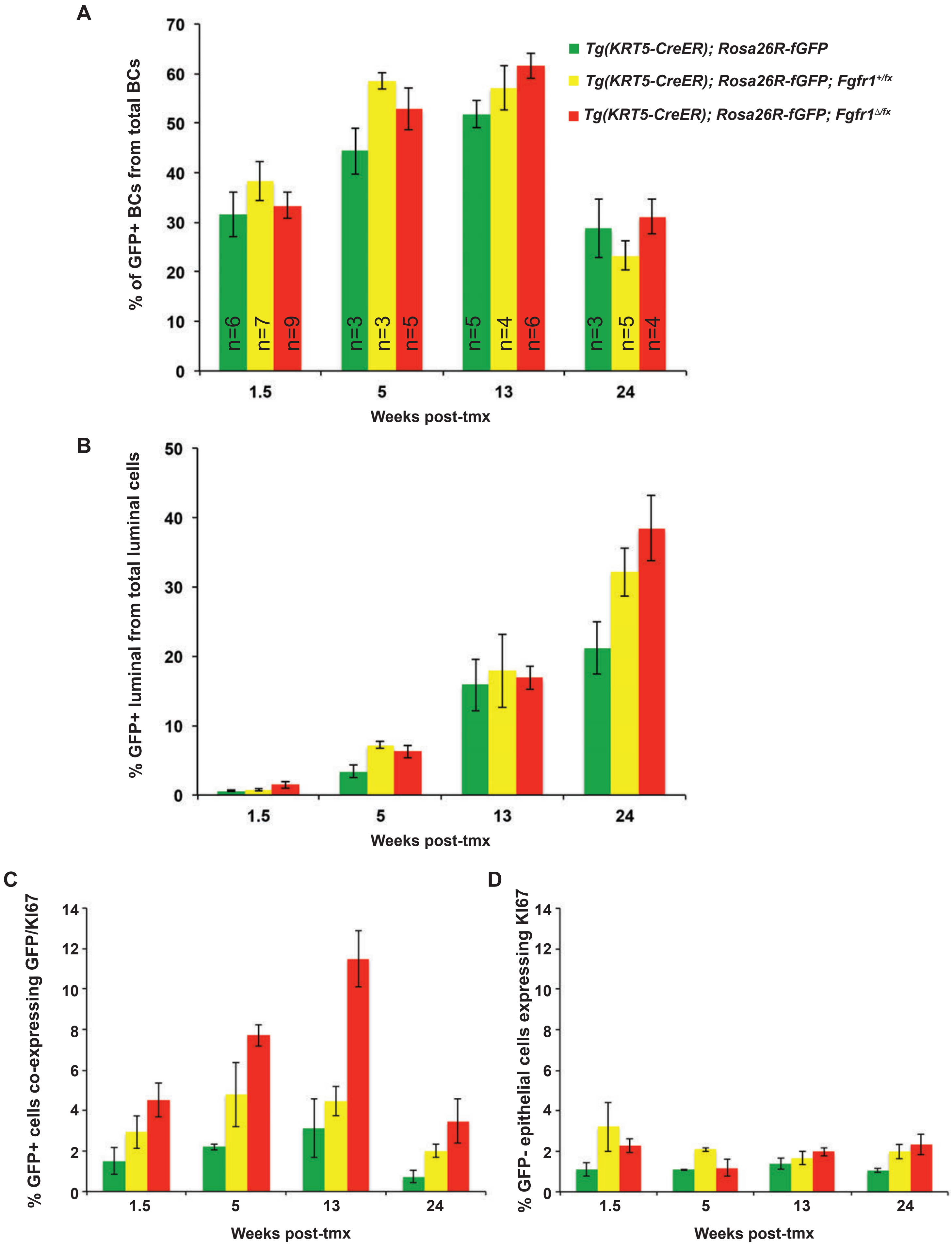

Balasooriya et al. Fig S3

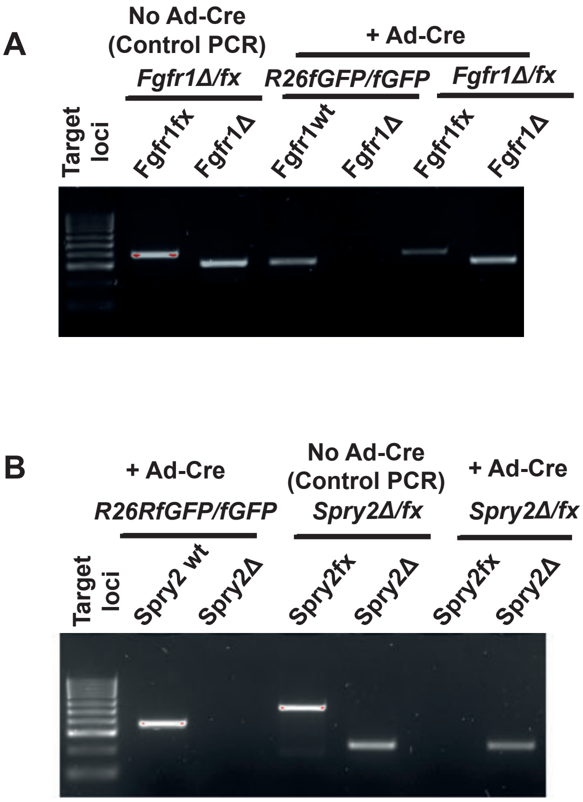

Balasooriya et al. Figure S4

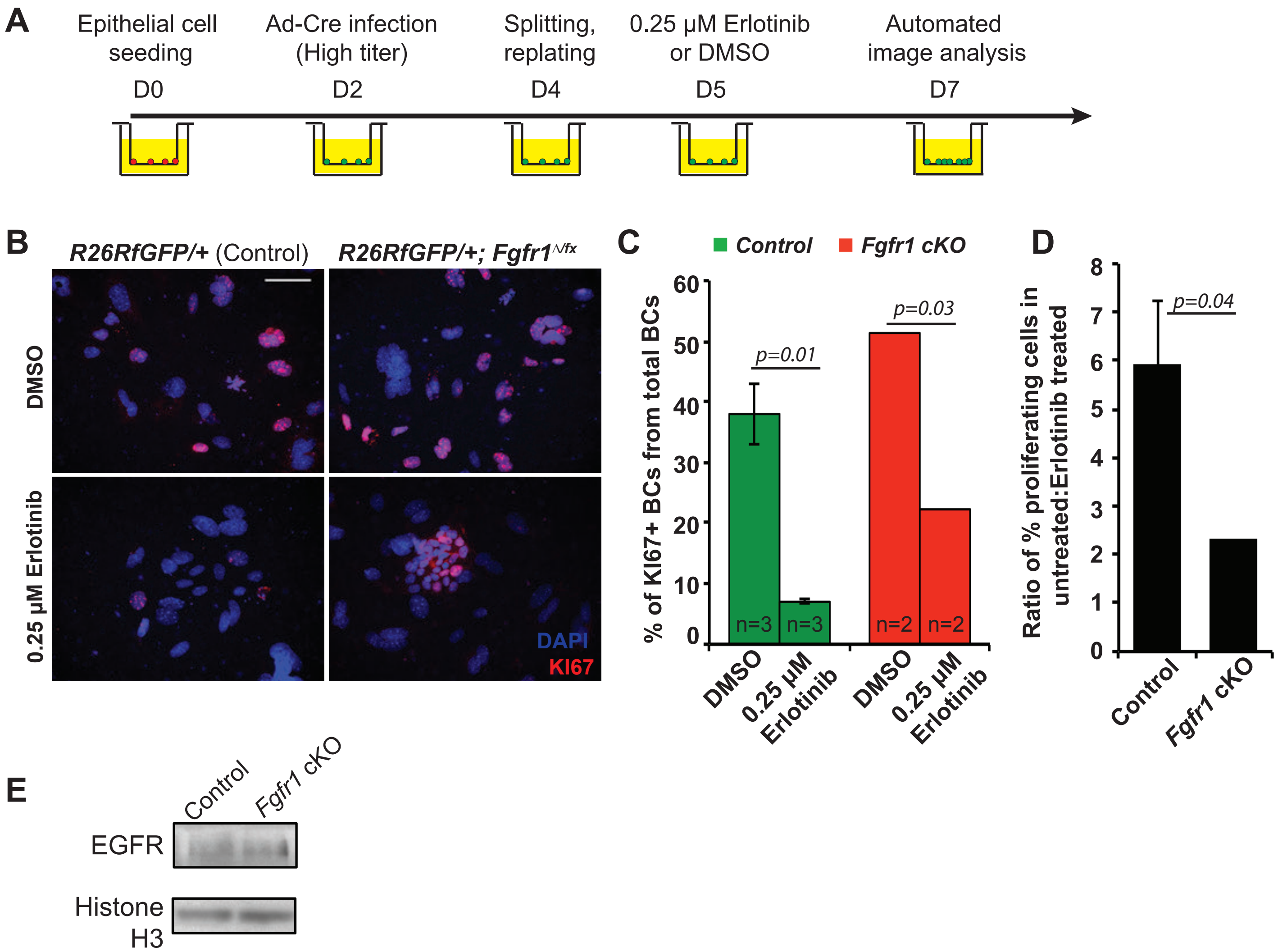

Balasooriya et al. Figure S5

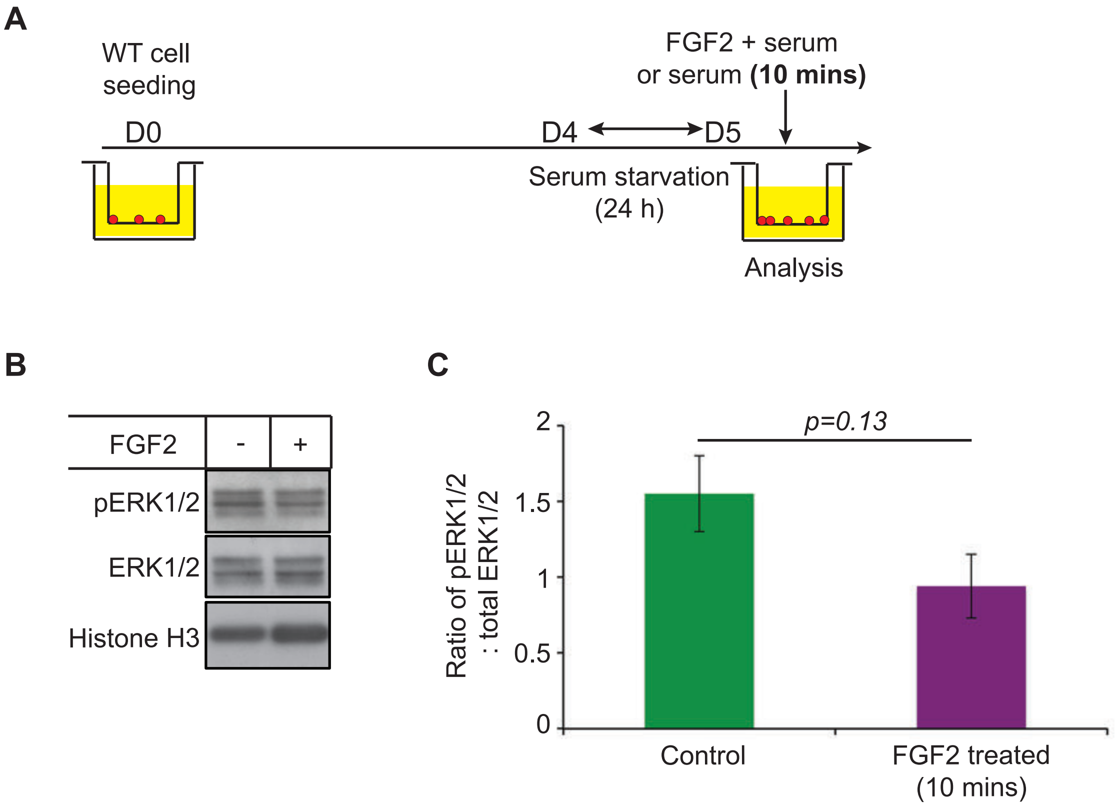

Balasooriya et al. Figure S6

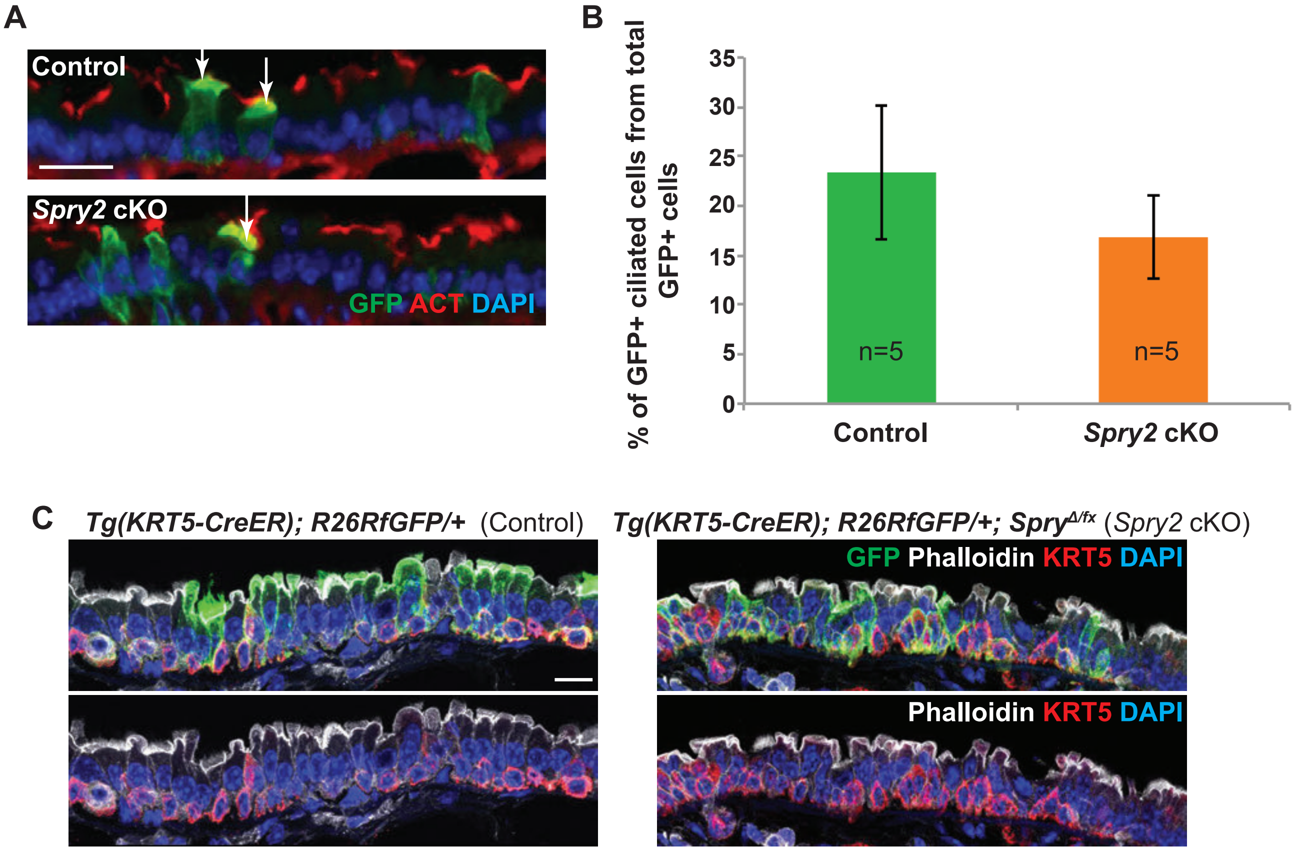

Figure S1, related to Figure 1. *Fgfr1* and *Spry2* are expressed in tracheal basal cells. (A) RT-PCR for components of the FGFR signalling pathway in the steady-state adult whole trachea, microdissected epithelium, or mesenchyme. There is widespread expression of mRNA for all receptors (except *Fgfr4* which was undetectable), all *Spry* signalling inhibitors and all tested FGF ligands. (B) Sorting of basal, secretory, and ciliated cells on surface expression of GSIβ4 lectin, SSEA1 and CD24 respectively. EpCAM is a marker of all airway epithelial cells. (C) RT-qPCR for *p63*, *Scgb1a1* and *Foxj1* in the sorted populations, normalised to *Ppia* levels, confirms basal, secretory and ciliated cell identity. Bars=SEM. (D) FGFR1 protein is expressed throughout the epithelium in wild-type tracheal epithelial sections. Green: FGFR1; red: T1α (basal cells); blue: DAPI (nuclei). FGFR1 can be seen clearly in the basal cells (arrowhead in inset) and the apical region of the luminal cells (arrows). (E) SPRY2 protein is expressed throughout the epithelium. Green: T1α (basal cells); Red: SPRY2; blue: DAPI (nuclei). (F) *Fgfr1* or *Spry2* mRNA in situ hybridisation in the steady-state adult trachea showing wide-spread epithelial expression. Scale bars = 50 μm D; 100 μm E, F.

Figure S2, related to Figures 1 and 2. *Fgfr1* heterozygous basal cells display a similar phenotype to conditional knock-out basal cells. (A-D) Graphs to show percentage of the total T1α$^+$ BCs that are also GFP$^+$ (A); percentage of the total T1α$^-$ luminal cells that are also GFP$^+$ (B); percentage of the total GFP$^+$ cells that are also KI67$^+$ (C) and percentage of the total GFP$^-$ epithelial cells that are also KI67$^+$ (D) in control, *Fgfr1* conditional heterozygous and cKO trachea. Note that there is no increase in proliferation of GFP$^-$ epithelial cells (compare D with C) suggesting that there are no widespread cell non-autonomous proliferation phenotypes in the mutant tracheae. Error bars = SEM.

Figure S3, related to Figures 3 and 6. Recombination of *Fgfr1*^{fx} and *Spry2*^{fx} in vitro. (A, B) Genomic PCR of *Fgfr1*$^{\Delta/fx}$ (A) and *Spry2* $^{\Delta/fx}$ (B) and control basal cells following Ad-Cre infection in vitro. Note the partial deletion of *Fgfr1*^{fx} (compare lane 1 with lane 5 in A), but the complete deletion of *Spry2*^{fx} (compare lane 3 with lane 5 in B).

Figure S4, related to Figure 3. *Fgfr1* cKO basal cells are more resistant to EGFR signalling inhibition. (A) Schematic of in vitro experiment. High titer Ad-Cre was used to delete floxed alleles, followed by passaging to provide internal controls for each biological replicate and 0.25 μM Erlotinib or DMSO treatment. Cells were scored by automated image analysis using a Fiji plug-in and thus analysis was independent of possible human bias. (B) Wild-type and *Fgfr1* cKO cells day 7 post-plating. Red: KI67 (proliferating cells); blue: DAPI (nuclei). Scale bar = 50 μm. (C) Percentage of proliferating (KI67$^+$) cells in each condition. (D) Ratio of the percentage of proliferating cells in DMSO : Erlotinib samples in control and *Fgfr1* cKO genotypes. Bars = SEM. (E) Representative western blots from control and *Fgfr1* cKO cells showing EGFR and Histone H3. No differences in EGFR levels were detectable.

Figure S5, related to Figure 3. FGF2 stimulation of wild-type basal cells results in a small drop in pERK1/2 levels. (A) Experimental time course. Wild type basal cells were serum-starved and then stimulated for 10 minutes with FGF2 and serum, or serum alone. (B) Representative western blots from control and FGF2-treated cells showing pERK1/2; total ERK1/2; Histone H3. (C) Quantification of protein in (B) normalised to loading control. Bars = SEM. There is a reproducible, but not statistically significant, decrease in the ratio of p : total ERK1/2 following FGF2 treatment.

Figure S6, related to Figure 5. Ciliated cell differentiation and epithelial organisation are normal in the *Spry2* conditional knock-outs. (A) Representative sections from control *Tg(KRT5-CreER); Rosa26R*$^{fGFP/+}$ and cKO *Tg(KRT5-CreER); Rosa26R*$^{fGFP/+}$*; Spry2*$^{\Delta/fx}$ tracheas at 13 weeks post-tmx. Green: GFP (*Rosa* reporter); red: acetylated tubulin (cilia); blue: DAPI (nuclei). Scale bar =20 μm. (B) Graph to show percentage of the total GFP$^+$ luminal cells that are also acetylated tubulin$^+$ at 13 weeks post-tmx. Bars = SEM. (C) Representative confocal z-projections of tracheal sections from control *Tg(KRT5-CreER); Rosa26R*$^{fGFP/+}$ and cKO *Tg(KRT5-CreER); Rosa26R*$^{fGFP/+}$*; Spry2*$^{\Delta/fx}$ animals at 13 weeks post-tmx. Green: GFP (*Rosa* reporter); red: KRT5 (basal cells); white: Phalloidin (F-actin); blue: DAPI (nuclei). Scale bar = 10 μm.

Supplemental Experimental Procedures

Mice

All experiments were approved by University of Cambridge local ethical review committees and conducted according to Home Office project licenses PPL80/2326 and 70/812. Mouse strains *Fgfr1*^{fx} (Xu et al., 2002), *Tg(KRT5-CreER)* (Rock et al., 2009), *Rosa26R-fGFP* (Rawlins et al., 2009), *Spry2*^{fx} (Shim et al., 2005) have all been described previously. Animals heterozygous for *Fgfr1*$^{\Delta/+}$ and *Spry2*$^{\Delta/+}$ were generated by crossing the respective floxed alleles to *Zp3-Cre* (de Vries et al., 2000). All transgenic strains were maintained on a C57Bl/6J background (at least N4 back-crosses) and a mixture of males and females were used for all experiments. No gender-specific differences were observed. All animals used for experiments were >8 weeks old. Wild-type C57Bl/6J inbred mice were used for RT-PCR, RT-qPCR, mRNA in situ hybridisation and FGF2 treatment.

Tamoxifen administration

Adult (>8 week) animals were injected intraperitoneally four times, every other day, with 0.2 mg / gram body weight tamoxifen.

Tracheal epithelial cell culture

Tracheal cells were isolated following published methods (Rock et al., 2009). Briefly, trachea were cut into small pieces and incubated in 50% Dispase II (Gibco, 16 U/ml) 20 minutes room temperature. Epithelial sheets were isolated by manual dissection and dissociated to single cells using 0.1% trypsin/EDTA. Unless otherwise stated, $5x10^4$ cells in 0.5ml MTEC/+ media (You et al., 2002) were plated on collagen-coated 12-well tissue culture inserts (BD Falcon, 353180) and grown to confluence. Differentiation was induced in confluent cultures by removal of medium from the insert and addition of MTEC serum free media to the outer chamber. Adeno-Cre (University of Iowa, Gene Transfer Vector Core) was incubated at the desired multiplicity of infection (MOI 7 for scattered recombination, MOI 2500 for complete recombination; vector pfu $1x10^6$) for 8 hours followed by washing in MTEC/+ media. Recombinant mouse FGF2 (Wellcome Trust/MRC Stem Cell Institute Tissue Culture Facility) was used at 100 ng/ml. Erlotinib (Sigma) was used at 0.25 μM. All in vitro experiments were preformed in triplicate unless otherwise stated.

Immunostaining

For protein detection tracheae were fixed in 4% paraformaldehyde at 4°C for 4 hours. Samples were washed in PBS, sucrose protected, embedded in OCT (Optimum Cutting Temperature Compound, Tissue Tek) and sectioned at 6 μm. For immunostaining of basal cell primary cultures, tissue culture inserts were washed in PBS and fixed 10 minutes in 4% paraformaldehyde at room temperature and cells were permeabilized in 0.3% triton X-100 in PBS. The following primary antibodies were used: acetylated tubulin (mouse, 1:3000, Sigma, T7451), E-cadherin (rat, 1:3000, Invitrogen, 13-1900), pERK1/2 (rabbit, 1:200, Cell signalling, 4695), FGFR1 (rabbit, 1:200, Novus Biologicals, NBP1-20067), FOXJ1 (mouse, 1:200, eBioscience, clone 2A5, 14-9965-80), GFP (chick, 1:1000, AbCam, AB13970), KRT5 (rabbit, 1:500, Covance, PRB-160P), KI67 (mouse, 1:200, BD, 550609), SCGB1A1 (goat, 1:400, Santa Cruz, sc9772), SPRY2 (rabbit, 1:200, Abcam, Ab50317), T1α (1:1000, DSHB, 8.1.1). Antigen retrieval was by boiling in 10mM sodium citrate, pH 8 for KI67, pH 6.5 for anti-pERK1/2, and pH 6 for FOXJ1. Secondary antibodies (1:2000 dilution) were Alexa Fluor conjugated from Life Technologies. DNA was stained with Dapi (Sigma) and F-actin with Phalloidin-568 (Life Technologies). Slides were mounted in Fluoromount (Sigma).

mRNA in situ hybridisation

For mRNA detection tracheas were fixed in 4% paraformaldehyde at 4°C overnight. Samples were washed in PBS, sucrose protected, embedded in OCT (Optimum Cutting Temperature Compound, Tissue Tek) and sectioned at 10 μm. DIG-labelled anti-sense mRNA probes to *Fgfr1* (EST ID: AW495528) or *Spry2* (Minowada et al., 1999) were prepared using standard methods (DIG RNA labelling Kit, Roche, 11175025910) and hybridised to tissue sections overnight at 65°C in a buffer containing 50% formamide. Following stringent post-hybridisation washes in 0.2x SSC, slides were blocked and incubated with Alkaline Phosphatase (AP) coupled anti-DIG (1:2000 – Roche 12930023). AP activity was visualized using BCIP (Roche, 1383213)/ NBT (Roche, 1383213). Sections were mounted in 70% glycerol and imaged in a Zeiss AxioImager compound microscope.

Microscopy and Image scoring

Slides were imaged on a Zeiss AxioImager compound microscope, or either an Olympus FV1000 or Leica Sp8/Sp5 confocal microscope where stated. Most experiments were scored manually in Fiji (ImageJ). For cryosections every epithelial cell along the entire proximal to distal length of a longitudinal section from the centre of the trachea was scored. Basal cells were scored as either T1α$^+$ or KRT5$^+$ with an apical surface which

did not extend to the lumen. Scoring of other cell types was based on markers as described in the text. For cultured cells, at least three random fields of view from each insert were scored. To assess cell density in control and *Spry2* cKO tracheas, straight lines were drawn along the basement membrane in regions with large patches of GFP$^+$ cells using Fiji and the number of Dapi$^+$ nuclei along the line was scored. For the Erlotinib experiments an automated scoring system was employed. Cells were segmented on the basis of DAPI$^+$ nuclei and the KI67 fluorescence intensity was measured using the Gurdon Institute Imaging Facility's Fiji plugin, ObjectScan. Cells were scored as KI67$^+$ by the plug-in if their fluorescence intensity exceeded a defined threshold.

RT-PCR

Samples were enriched for epithelium or mesenchyme using Dispase digestion and manual peeling as described for tracheal epithelial cell culture. Total RNA was extracted using Trizol reagent and cDNA was synthesised using Superscript III reverse transcriptase (Life Technologies). Primer sequences:
Fgf1-F: TTGACCAATGCTGAGCCTAC
Fgf1-R: AGAGCAGTTTGGGCTTTTTG
Fgf2-F: CAACCGGTACCTTGCTATGA
Fgf2-R: ACTGCCCAGTTCGTTTCAGT
Fgf7-F: CCATGAACAAGGAAGGGAAA
Fgf7-R: TTGACAGGAATCCCCTTTTG
Fgf10-F: CCATGAACAAGAAGGGGAAA
Fgf10-R: CTCTCCTGGGAGCTCCTTTT
Fgfr1 IIIb-F: CCAACCTCTAACCGCAGAAC
Fgfr1 IIIb-R: TCGTCGTCGTCATCATCTTC
Fgfr1 IIIc-F: TCACAGCCACTCTCTGCACT
Fgfr1 IIIc-R: CCAGTTGATGCTCTGCACAT
Fgfr2 IIIb-F: ACCGAGAAGATGCATGGAGAAGC
Fgfr2 IIIb-R: TAAGGCTCCAGTGCTGGTTT
Fgfr2 IIIc-F: GCTAGGACGGTAGACA
Fgfr2 IIIc-R: TCTGGTTGCTCCTGTTCTCA
Fgfr3 IIIb-F: TGAAGCACGTGGAAGTGAAC
Fgfr3 IIIb-R: GGTGGCTCGACAGAGGTACT
Fgfr3 IIIc-F: TGAAGCACGTGGAAGTGAAC
Fgfr3 IIIc-R: TCTAGCTCCTTGTCGGTGGT
Fgfr4-F: GGAGGAGCTCTTCTCACTGC
Fgfr4-R: CAGGACCTTGTCCAGAGCTT
Spry1-F: GGCCTATTAGGACGGTCTCC
Spry1-R: CAGTGGGACTGTGAACAGGA
Spry2-F: ACATCGCTGGAAGAAGAGGA
Spry2-R: CAGGTCTTGGCAGTGTGTTC
Spry3-F: TTCTGCATGGTCATCATTTG
Spry3-R: GGCACTTGCGGTGATTTA
Spry4-F: GGTTCGGGGATTTACACAGA
Spry4-R: CATGACTGAGCTGGGATTCA
K5-F: TCCAGAACGCCATTGCTGAAG
K5-R: CCGTAGCCAGAAGAGACACTGTTTG
VE-Cadherin-F: AAGCAGAACCTGACCTGGAA
VE-Cadherin-R:GTGAGGGGCA ATGACAATCT
β-Actin-F: CCCTGAAGTACCCCATTGAA
β-Actin-F: CTTTTCACGGTTGGCCTTAG.

RT-qPCR

Primary tracheal epithelial cells were isolated as for cell culture. Cells were resuspended in FACS buffer (2.0% FBS in PBS). Cells were stained with EpCAM-PECy7 (1:100; 25-5791-80, eBiosciences), GSIβ4-FITC (1:100; L2895, Sigma), SSEA1-Alexa Fluor® 647 (1:50; 125608, BioLegend), and CD24-PE (1:75; 553262, BD Pharmingen) for 30 minutes on ice (Zhao et al., 2014). Cells were sorted using a fluorescence-activated cell sorting MoFlo flow cytometer. Wild-type basal cells were sorted as EpCAM$^+$, GSIβ4 lectin$^+$ positive; secretory cells as EpCAM$^+$, SSEA1$^+$; ciliated cells as EpCAM$^+$, CD24$^+$. GFP$^+$ basal cells from control and *Fgfr1* cKO tracheae were sorted using endogenous GFP fluorescence from *Rosa26R-fGFP* and GSIβ4-647 (1:100, 132450, Life Technologies). Total RNA was extracted using Qiagen RNEasy Mini Kit and cDNA was synthesised using Superscript III reverse transcriptase (Life Technologies). Taqman gene expression assays (Life Technologies) were used for expression analysis: *Fgfr1* (Mm00438930_m1); *Foxj1* (Mm01267279_m1); *p63*

(Mm00495791_m1); *Ppia* (Mm02342429_g1); *Scgb1a1* (Mm00442046_m1); *Spry2* (Mm00442344_m1).

Immunoblot analysis

Cultured cells were collected in Cell Extraction Buffer (Invitrogen, FNN0011) with 1x cocktail protease inhibitor (Roche 04693116001) and PMSF (Sigma, P7626). Protein concentration of the lysate was measured by Nanodrop and proteins separated on 10 or 12% SDS-PAGE gels before transfer onto Millipore Immobilon-P PVDF Membrane (Merck Millipore, IPVH00010). Membranes were blocked with 5% dried skim milk and probed with p-AKT(S473) (Cell Signalling, 3787, 1:3000), pan-AKT (Cell Signalling, 4691, 1:1000), dpERK1/2 (Cell Signalling, 4370, 1:300), ErRK1/2 (Cell Signalling, 4695, 1: 300), SPRY2 (Abcam, ab50317, 1:300), SOX2 (Abcam, Cat: ab97959, 1:3000), H3 (Abcam, ab39655, 1:10000), EGFR (Millipore, 04-290, 1:300) primary antibodies. Detection was with HRP-conjugated secondaries goat anti-rabbit IgG H&L (Abcam, ab97051, 1:10000), goat anti-mouse IgG H&L (Abcam, ab97023, 1:10000) and enhanced chemiluminescense (Thermo Scientific, PI-32109). Representative blots are shown. Quantitation is based on the analysis of protein from three biological replicates which were separated on the same polyacrylamide gel. Band intensity was analysed in Fiji normalised to the loading control.

Statistics

All p-values shown were obtained using an unpaired two-tailed student's t-test with unequal variance.

REFERENCES

de Vries, W.N., Binns, L.T., Fancher, K.S., Dean, J., Moore, R., Kemler, R., and Knowles, B.B. (2000). Expression of Cre recombinase in mouse oocytes: a means to study maternal effect genes. Genesis *26*, 110-112.

Minowada, G., Jarvis, L.A., Chi, C.L., Neubuser, A., Sun, X., Hacohen, N., Krasnow, M.A., and Martin, G.R. (1999). Vertebrate Sprouty genes are induced by FGF signaling and can cause chondrodysplasia when overexpressed. Development *126*, 4465-4475.

Rawlins, E.L., Okubo, T., Xue, Y., Brass, D.M., Auten, R.L., Hasegawa, H., Wang, F., and Hogan, B.L. (2009). The role of Scgb1a1+ Clara cells in the long-term maintenance and repair of lung airway, but not alveolar, epithelium. Cell stem cell *4*, 525-534.

Rock, J.R., Onaitis, M.W., Rawlins, E.L., Lu, Y., Clark, C.P., Xue, Y., Randell, S.H., and Hogan, B.L. (2009). Basal cells as stem cells of the mouse trachea and human airway epithelium. Proc Natl Acad Sci U S A *106*, 12771-12775.

Shim, K., Minowada, G., Coling, D.E., and Martin, G.R. (2005). Sprouty2, a mouse deafness gene, regulates cell fate decisions in the auditory sensory epithelium by antagonizing FGF signaling. Dev Cell *8*, 553-564.

Xu, X., Qiao, W., Li, C., and Deng, C.X. (2002). Generation of Fgfr1 conditional knockout mice. Genesis *32*, 85-86.

You, Y., Richer, E.J., Huang, T., and Brody, S.L. (2002). Growth and differentiation of mouse tracheal epithelial cells: selection of a proliferative population. Am J Physiol Lung Cell Mol Physiol *283*, L1315-1321.

Zhao, R., Fallon, T.R., Saladi, S.V., Pardo-Saganta, A., Villoria, J., Mou, H., Vinarsky, V., Gonzalez-Celeiro, M., Nunna, N., Hariri, L.P*., et al.* (2014). Yap Tunes Airway Epithelial Size and Architecture by Regulating the Identity, Maintenance, and Self-Renewal of Stem Cells. Dev Cell *30*, 151-165.
